# Pathway and mechanism of tubulin folding mediated by TRiC/CCT along its ATPase cycle revealed using cryo-EM

**DOI:** 10.1038/s42003-023-04915-x

**Published:** 2023-05-16

**Authors:** Caixuan Liu, Mingliang Jin, Shutian Wang, Wenyu Han, Qiaoyu Zhao, Yifan Wang, Cong Xu, Lei Diao, Yue Yin, Chao Peng, Lan Bao, Yanxing Wang, Yao Cong

**Affiliations:** 1 State Key Laboratory of Molecular Biology, National Center for Protein Science Shanghai, Shanghai Institute of Biochemistry and Cell Biology, Center for Excellence in Molecular Cell Science, Chinese Academy of Sciences, 200031 Shanghai, China; 2 University of Chinese Academy of Sciences, 100049 Beijing, China; 3 State Key Laboratory of Cell Biology, Shanghai Institute of Biochemistry and Cell Biology, Center for Excellence in Molecular Cell Science, Chinese Academy of Sciences, 200031 Shanghai, China; 4 National Facility for Protein Science in Shanghai, Zhangjiang Lab, Shanghai Advanced Research Institute, CAS, 201210 Shanghai, China

**Keywords:** Cryoelectron microscopy, Chaperones

## Abstract

The eukaryotic chaperonin TRiC/CCT assists the folding of about 10% of cytosolic proteins through an ATP-driven conformational cycle, and the essential cytoskeleton protein tubulin is the obligate substrate of TRiC. Here, we present an ensemble of cryo-EM structures of endogenous human TRiC throughout its ATPase cycle, with three of them revealing endogenously engaged tubulin in different folding stages. The open-state TRiC-tubulin-S1 and -S2 maps show extra density corresponding to tubulin in the cis-ring chamber of TRiC. Our structural and XL-MS analyses suggest a gradual upward translocation and stabilization of tubulin within the TRiC chamber accompanying TRiC ring closure. In the closed TRiC-tubulin-S3 map, we capture a near-natively folded tubulin—with the tubulin engaging through its N and C domains mainly with the A and I domains of the CCT3/6/8 subunits through electrostatic and hydrophilic interactions. Moreover, we also show the potential role of TRiC C-terminal tails in substrate stabilization and folding. Our study delineates the pathway and molecular mechanism of TRiC-mediated folding of tubulin along the ATPase cycle of TRiC, and may also inform the design of therapeutic agents targeting TRiC-tubulin interactions.

## Abstract (web summary)

Cryo-EM and XL-MS analyses reveal the conformational landscape of TRiCmediated tubulin folding along TRiC’s ATPase cycle and the interaction sites between tubulin and the closed TRiC chamber.

## Introduction

Chaperonins are protein-folding nanomachines that play an essential role in maintaining cellular homeostasis and are important in all kingdoms of life, and their dysfunction is closely related to cancer and neurodegenerative diseases^1–4^. Chaperonins provide a cage-like environment for proteins to fold in isolation, unimpaired by aggregation, and in some cases actively modulate the folding pathway of the encapsulated protein^5–8^. The eukaryotic group II chaperonin TRiC/CCT assists the folding of ~10% of cytosolic proteins, including the key cytoskeletal proteins actin and tubulin, cell cycle regulator CDC20, and VHL tumor suppressor^9–16^. TRiC is the most complex chaperone system identified to date. It has a double-ring structure, and each ring consists of eight paralogous subunits (denoted as CCT1–CCT8) arranged in a specific order^17–22^. Each TRiC subunit consists of three domains: the substrate-recognition apical domain (A domain) and the ATP-binding equatorial domain (E domain) linked by the intermediate domain (I domain). TRiC-mediated folding of the substrate is closely related to the ATP-driven TRiC conformational cycle^23–26^. TRiC has been proven to display subunit specificity in the complex assembly, ATP consumption, and ring closure^17,20,27,28^.

In the past two decades, constant efforts have been made to capture the TRiC-substrate binary complexes so as to reveal the mechanism of TRiC-assisted substrate folding for diverse substrates^8,10,12,16,29–32^.Tubulin, the building block of the microtubule, is the in vivo obligate substrate of TRiC^16,33–36^. Highly conserved α-β-tubulin heterodimers assemble into dynamic microtubules^37^, which are ubiquitous cytoskeletal polymers essential for the survival of all eukaryotic cells. Microtubules are involved in a wide range of cellular functions, from cell motility and intracellular transport to, most fundamentally, cell division^35,38–42^. Tubulin (~50 kDa) contains three domains: the GTP-binding N domain, the I domain containing the site that binds the chemotherapeutic Taxol, and the C domain responsible for interacting with microtubule-binding proteins. There have been several structural studies on the TRiC-tubulin complex in apo and ATP-bound states^10,16,32^, in addition to a recent study presenting a closed-state TRiC cryo-EM structure engaged with a tubulin folding intermediate to a resolution of 3.0 Å and an open-state TRiC structure with a central density located in the inter-ring septum^43^, together paving the way toward a better understanding of TRiC-mediated tubulin folding. However, a thorough picture of TRiC-directed tubulin folding along the ATPase cycle of TRiC remains obscure. Moreover, it has been reported that the small molecule I-Trp can disrupt constitutively associated β-tubulin/TRiC, and hence cause apoptotic cell death, indicating that targeting the TRiC/tubulin complex could serve as a novel chemotherapeutic strategy^44,45^.

To describe the TRiC-directed tubulin folding pathway, we determined an ensemble of cryo-EM structures of endogenous human TRiC (hTRiC) throughout its ATP-driven conformational cycle, with three of them displaying endogenously engaged tubulin. The open-state TRiC-tubulin-S1 and -S2 maps showed extra density, corresponding to tubulin, in the cis-ring chamber of TRiC. Strikingly, in the closed-state TRiC-tubulin-S3 structure resolved to 3.1-Å resolution, we captured a near-natively folded tubulin and determined atomic details of the interaction between tubulin and TRiC. We also found distinct roles played by the C- and N-terminal tails (abbreviated as C-/N-termini) of TRiC in substrate stabilization/folding and TRiC allosteric coordination, respectively, with the N-terminus of CCT8 possibly contributing to tubulin folding in the opposite ring. We also obtained a TRiC-ADP structure with a substate being released from the chamber. Collectively, our study captured a thorough picture of the pathway and molecular mechanism of TRiC-mediated tubulin folding along the TRiC ATPase cycle.

## Results

### Cryo-EM structure of TRiC in complex with tubulin

**Fig. 1:**
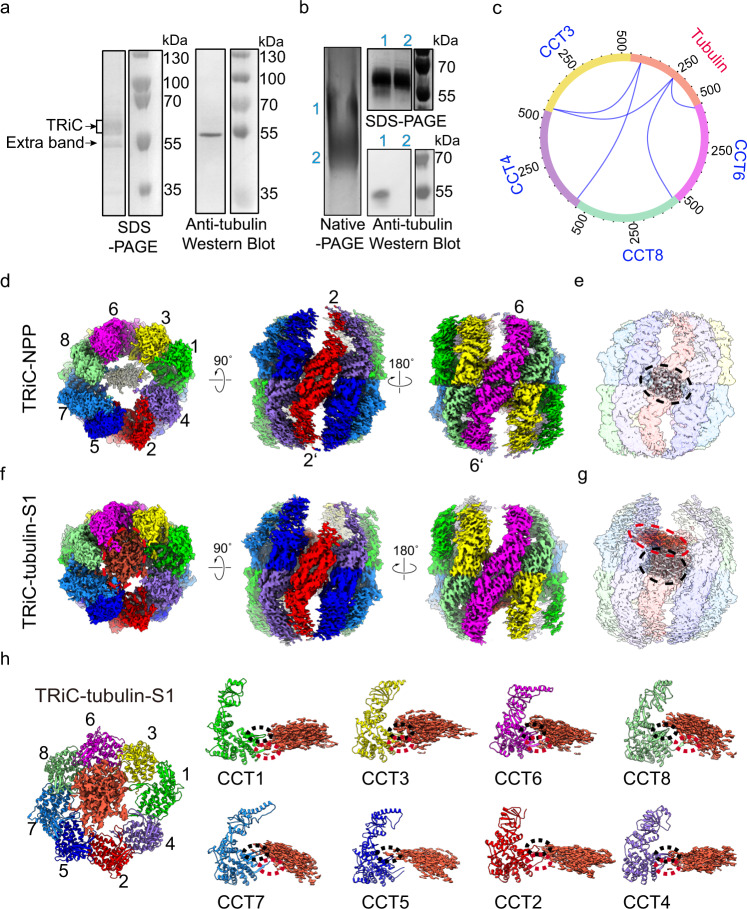
Cryo-EM structures of the endogenously purified TRiC with associated tubulin. **a** SDS-PAGE of the endogenous human TRiC purified from HEK293F cells. This gel suggested the presence of an extra associated protein at ~50 kDa, which was proved to be tubulin by Western blot. **b** Native gel analysis of TRiC, showing two bands, labeled as 1 and 2 (left), which were excised and loaded into, respectively, an SDS-PAGE system (upper right). This SDS-PAGE analysis suggested that both bands contained TRiC oligomer, and there is an additional band was observed in lane 1. This additional band contained tubulin, as shown using western blotting (lower right). **c** XL-MS analysis of the endogenously purified TRiC with associated tubulin. Detected TRiC-tubulin cross-links are shown as blue lines. **d** End-on and side views of TRiC-NPP, with the different subunits in different colors and labeled. This subunit color scheme is followed in subsequent figures. **e** Unstructured N- and C-terminal tail density (in gray, indicated by a black dashed ellipsoid) of TRiC subunits located between the two equators in TRiC-NPP (transparent density). **f**, **g** Cryo-EM map of TRiC-tubulin-S1 (**f**), revealing extra density (shown in red and indicated by a red dashed ellipsoid) within the cis-ring chamber of TRiC (**g**) adjacent to the unstructured TRiC tail density (gray). **h** Overall binding location of tubulin (red density) in the cis-ring of TRiC-tubulin-S1 (colored ribbon) (left), and the association of tubulin with the E domain of each TRiC subunit, including with its resolved portion of C-terminus (indicated by dotted red ellipsoid) and stem loop (dotted black ellipsoid) (right panels).

We purified endogenous human TRiC from HEK293F cells (Supplementary Fig. 1a; hereafter, “TRiC” refers to human TRiC unless otherwise noted), and our NADH-coupled enzymatic assay result further validated the ATPase activity of the purified TRiC (Supplementary Fig. 1b). Moreover, the sodium dodecyl sulfate-polyacrylamide gel electrophoresis (SDS-PAGE) analysis indicated the presence of an extra associated protein at ~50 kDa (Fig. 1a), which was suggested to be mainly β-tubulin (TUBB5) according to the mass spectrometry (MS) analysis (Supplementary Table 1). The PSM value from the MS analysis, indicating the relative abundance of a certain protein^46^, showed that the abundance of β-tubulin was comparable to that of an individual TRiC subunit. The presence of tubulin was further confirmed using native electrophoresis combined with western blot and chemical cross-linking-coupled mass spectrometry (XL-MS) analysis (Fig. 1b, c and Supplementary Table 2). Our XL-MS analysis detected five cross-links between tubulin and CCT3/4/6/8 subunits (Fig. 1c). Taken together, these data indicated a co-existence of TRiC with tubulin.

We then performed cryo-EM study on the endogenously purified TRiC sample (Supplementary Fig. 1c). Two cryo-EM maps, including an open-state TRiC with an empty chamber (61.6% of the population), and another open-state TRiC but displaying an extra density in one chamber (38.4% of the population), were determined to resolutions of 3.1 Å and 4.1 Å, respectively (Fig. 1d–g and Supplementary Fig. 2). Interestingly, although we performed buffer exchange multiple times to remove remaining nucleotide in the final stage of purification, CCT3/6/8 subunits in both maps appeared to have bound nucleotide density (Supplementary Fig. 3e, f), which were suggested to be ADP according to an ADP/ATP ratio assay (Supplementary Fig. 3g), in line with our previous report on yeast TRiC^20^. Accordingly, these two open-state TRiC maps were also in the “nucleotide partially preloaded” (NPP) state. We then denoted the empty chamber open TRiC map as TRiC-NPP. The NPP state is equivalent to the “apo” state, the starting point in the conformational cycle of the chaperonin itself^25,47,48^.

Our TRiC-NPP map displayed a conformation similar to that of the available substrate-free open-state human TRiC map^49^, with a determined subunit assignment (Supplementary Fig. 4a). Both maps displayed several characteristic features known for open-state TRiC: (1) CCT1 being the most outward tilted subunit, a feature common for yeast, bovine, and human TRiCs^8,17,20,49^; (2) the A domain of CCT2 being quite disordered (Fig. 1d), also observed in open bovine TRiC^8,25^; (3) each ring displaying a tetramer-of-dimers pattern as in the open bovine TRiC^25^; and (4) the largest gap occurring between CCT1 and CCT4^50^ (Supplementary Fig. 4b). These features allowed us to assign the subunits for the TRiC-NPP map. We then built an atomic model for TRiC-NPP (Supplementary Fig. 3a, b). Further inspection of the TRiC-NPP structure revealed the characteristic V476-K484 insertion in the E domain of CCT1 (Supplementary Fig. 4c, d), corroborating our subunit assignment for this map.

Moreover, the dominantly populated (61.6%) TRiC-NPP map showed a chunk of density between the two equators blocking the two chambers (Fig. 1e), and this central density was also observed in previous bovine and human free TRiC open-state maps^8,49^ (Supplementary Fig. 4f, g). This density symmetrically contacts the N-/C-terminal extensions of CCT5/7 and CCT5’/7’ from both rings of TRiC (Supplementary Fig. 4e). Hence, we assigned the density as the unstructured N- and C-terminal tails of TRiC subunits. Moreover, the A/I domains of CCT2 hemisphere subunits (including CCT4/2/5/7)^22,29^ appeared less well resolved, also observed in the bovine and human open-state TRiC structures^8,25,29,49^, indicating an intrinsically dynamic nature in these regions, which was substantiated by a further 3D variability analysis (3DVA) using cryoSPARC^51^ (Supplementary Fig. 4h and Supplementary Video 1).

Importantly, for the other open-state TRiC map derived from the same dataset (Fig. 1f, g and Supplementary Fig. 2a), we attributed the extra density in the cis-ring chamber to trapped β-tubulin co-purified with TRiC, based on our biochemical and MS data (Fig. 1a–c, Supplementary Fig. 4i and Supplementary Tables 1 and 2). We denoted this map as TRiC-tubulin-S1, which was also in the NPP state with consecutive CCT3/6/8 subunits loaded with nucleotides in both rings (Supplementary Fig. 3f), indicating that TRiC can engage with the substrate in the NPP state. The TRiC conformation in the S1 map resembled that of our TRiC-NPP structure, so we made the same subunit assignment and built the atomic model (Supplementary Fig. 3c, d). Overall, substrate density appeared to locate right above the central N-/C-terminal tail density of TRiC, and associate with the E domain of every TRiC subunit, mainly with the C-terminus and stem loop of each of these subunits (Fig. 1h). A previous crystal structure of bovine TRiC-tubulin^16^ also revealed two nucleotides per ring in non-consecutive subunits, and that the tubulin forms contacts with the stem loops of three TRiC subunits.

### Tubulin translocation within the TRiC chamber accompanying TRiC conformational cycle

**Fig. 2:**
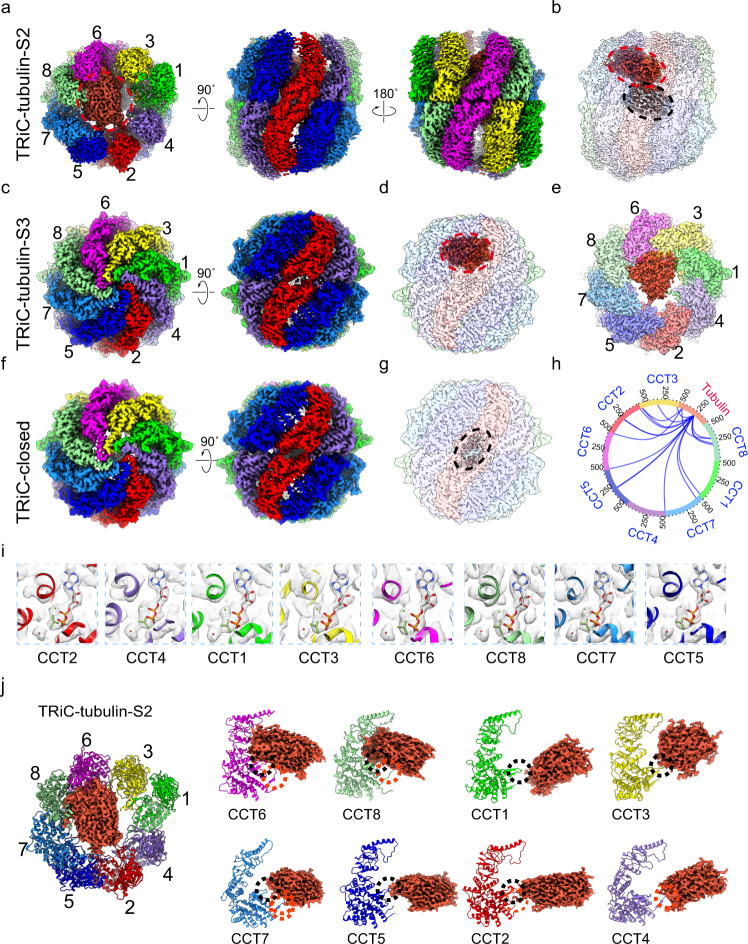
Cryo-EM structures of TRiC-tubulin in the presence of ATP-AlFx. **a**, **b** Cryo-EM map of TRiC-tubulin-S2 in the open ATP-binding state (**a**), revealing a tail density (in gray) between the two equators and a tubulin density (in red) in the cis-ring of TRiC (transparent density) (**b**). **c**, **d** Cryo-EM map of TRiC-tubulin-S3 in the closed ATP-hydrolysis transition state (**c**), revealing tubulin density in the cis-ring chamber of TRiC (**d**). **e** Top view of the TRiC-tubulin-S3 map with TRiC A domains omitted for easier visualization, showing the bound tubulin density, and indicating tubulin mainly associated with CCT6 hemisphere subunits (CCT1/3/6/8). **f**, **g** Cryo-EM map of TRiC-closed (**f**), revealing a mass of C-termini (black dashed ellipsoid) (**g**), related to Supplementary Fig. 5f. **h** XL-MS analysis of TRiC with endogenously associated tubulin in the presence of ATP-AlFx, showing only the detected TRiC-tubulin cross-links. **i** Nucleotide pocket region of each subunit of the TRiC-tubulin-S3 map. All eight subunits bound ADP-AlFx (stick model) and a magnesium ion (green ball), as well as a water molecule (red ball) in an attacking position. **j** Overall binding of tubulin (red density) in the cis-ring of the open TRiC-tubulin-S2. Tubulin was observed to contact all three domains of CCT6/8 and loosely contact the E domains, including the C-terminus (dotted red ellipsoid) and stem loop (dotted black ellipsoid), of the remaining subunits.

To further capture the TRiC-directed tubulin folding process along the TRiC ATPase cycle, we performed a cryo-EM study on TRiC in the presence of 1 mM ATP-AlFx (Supplementary Fig. 1d), an ATP-hydrolysis-transition-state analog that can trigger TRiC ring closure^26^ and has been used in many previous structural studies on TRiC and TRiC-substrate complexes^8,21,23–26,52,53^. In the resulting dataset, the majority (65.4%) of the particles were resolved to a closed-state TRiC map at 2.9 Å resolution, while the remaining (34.6%) particles were resolved to an open-state map at 4.2 Å resolution (Supplementary Fig. 5a–c). In the open-state map, obvious substrate density was observed in the cis-ring (denoted as TRiC-tubulin-S2, Fig. 2a). For the closed state, through focused classification in the chamber region, we obtained two maps at 3.1–3.2 Å resolution: one map showed tubulin density in the cis-ring chamber (termed TRiC-tubulin-S3, Fig. 2c–e and Supplementary Fig. 5a, b, d), and the other map showed no substrate in the chamber, but a central tail density in an orientation different from that in the open NPP, S1, and S2 states (denoted as TRiC-closed, discussed below) (Fig. 2f, g and Supplementary Fig. 5a, b, e, f). Moreover, we performed XL-MS analysis on the sample of TRiC with ATP-AlFx, and detected 17 cross-links between tubulin and TRiC subunits (Fig. 2h and Supplementary Table 3), further substantiating the notion that the captured substrate was indeed tubulin.

The overall TRiC conformation of the open-state S2 map was similar to that of the TRiC-NPP map (Supplementary Fig. 5g). We then followed the subunit assignments of TRiC-NPP to assign those of S2. For the closed-state S3 map, a characteristic kink feature in the CCT6 α-helical protrusion H8 and the unique insertions in CCT1/4/6 were clearly visualized (Supplementary Fig. 6a–c), facilitating the subunit assignment in the maps of the closed TRiC with similar features. We then built an atomic model for each of the S2, S3 and TRiC-closed maps (Supplementary Fig. 7a–f). We found that for these three structures, all subunits from both rings bound nucleotides (Supplementary Fig. 7g–i). For the S2 map, the nucleotide density was fitted well by the ATP model (Supplementary Fig. 7j), indicating that S2 was in the ATP-binding state. For the closed S3 and TRiC-closed structures, the nucleotide densities for all subunits matched ADP-AlFx and a magnesium ion very well, in addition to a water molecule attacking the γ-phosphate of nucleotide, involved in the formation of the ATP hydrolysis reaction center (Fig. 2i and Supplementary Fig. 7k). These features indicated that the two closed-state maps were of TRiC in the ATP-hydrolysis transition state^17,26^.

Indeed, the CCT2 subunit in the S2 map appeared less dynamic and better resolved than did that in the NPP-state S1 map (Figs. 1f and  2a), indicating that CCT2 may have been stabilized after it bound ATP. Accordingly, the substrate density in the S2 map, located above the central tail density of TRiC (Fig. 2b), appeared larger than that in the S1 map, with this difference attributed to ATP-binding-induced stabilization of TRiC (Figs. 1h and 2j). Notably, in TRiC-tubulin-S2, the substrate density was found to be closely associated with all three domains of CCT6/8 and to form loose contacts with the E domains of the other subunits (Fig. 2j). Compared with TRiC-tubulin-S1, it appeared that binding of ATP could drive tubulin to translocate from the E domains of all the subunits slightly up toward the A/I domains of TRiC, to converge more on the CCT6/8 subunits, while nevertheless remaining bound to all of the E domains (Fig. 2j).

### ATP-driven TRiC ring closure is the determinant step for tubulin folding

**Fig. 3:**
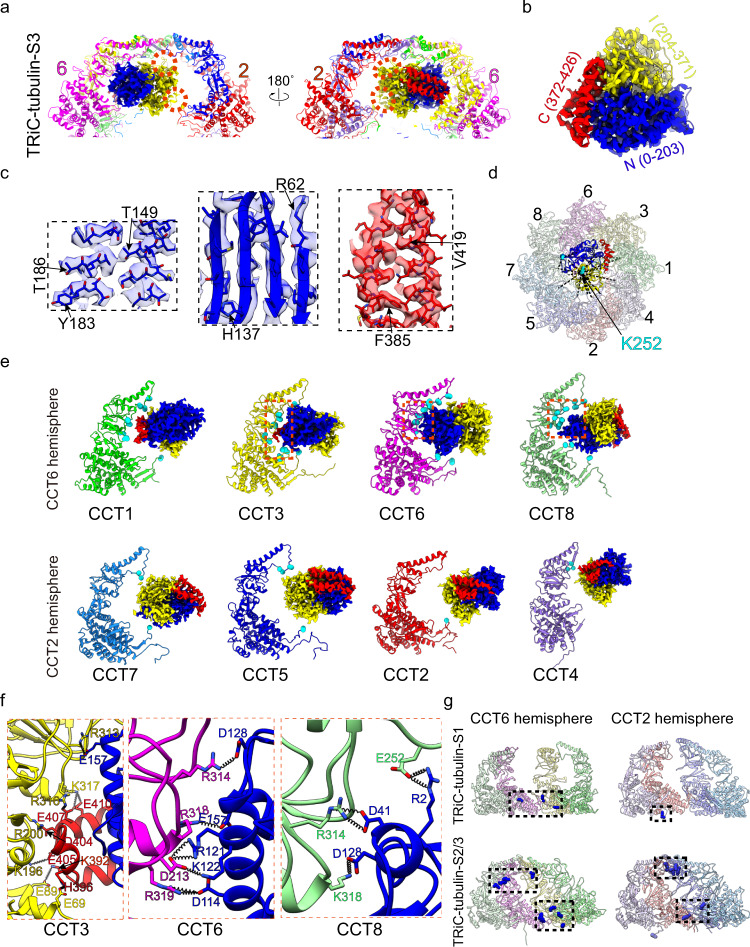
TRiC-tubulin-S3 structure showing a near-natively folded tubulin attached to the cis-ring of TRiC. **a** Enlarged views of the engaged tubulin within the TRiC chamber in the S3 state. The tubulin N/I/C domains were rendered in blue, yellow and red, respectively, with this color scheme followed throughout the figures. Red dotted circles indicate dynamic regions of the I domain of tubulin. **b**, **c** The resolved β-tubulin inside the cis-ring of TRiC-tubulin-S3 (**b**), and its high-resolution structural features (**c**). **d** Mapping of the detected cross-links made by tubulin K58 and K252 (cyan spheres) with the TRiC subunits in the cis-ring of TRiC-tubulin-S3. Note that every TRiC subunit made at least one such cross-link. **e** Ribbon diagram depictions of the association between each TRiC subunit and tubulin in S3, showing the close associations of the N/C domains of tubulin with CCT6 hemisphere subunits CCT1/3/6/8, and loose associations of the tubulin I domain with CCT2 hemisphere subunits CCT7/5/2/4. Cα atoms of the TRiC amino acid residues within 4 Å distance of tubulin were shown as cyan balls. **f** Magnified views of the regions indicated with red dotted frames in (**g**) to show the salt-bridge interactions formed between tubulin and CCT3/6/8. **g** XL-MS-analysis-derived sites on TRiC (blue spheres) cross-linked with tubulin and mapped onto the corresponding indicated TRiC structure. This analysis suggested a shift in the interaction locations induced by ATP-binding/hydrolysis.

Importantly, inspection of our TRiC-tubulin-S3 map revealed a well-resolved tubulin density in one chamber of TRiC, hanging underneath the TRiC dome predominantly attached to the CCT6 hemisphere subunits (including CCT1/3/6/8^22,29^) (Figs. 2d, e and  3a). The tubulin density appeared to resemble the conformation of native tubulin (Fig. 3b), and hence we defined the captured tubulin as being in the near-natively folded state. Overall, the N and C domains of tubulin were relatively well resolved with atomic details observed (Fig. 3c). While the majority of the I domain of tubulin was captured (Fig. 3b), a small portion facing the central chamber was less well resolved (Fig. 3a), implying an intrinsically dynamic nature for this region. Substantiating this speculation, our XL-MS analysis showed that tubulin I domain Lys252 formed cross-links with all subunits of TRiC, with the cross-linked Cα-Cα distances ranging from 20 to 61 Å (Fig. 3d), indicating a highly dynamic structure in this region.

Inspection of the S3 map showed that tubulin engaged with TRiC mainly through its N/C domains, forming intimate salt-bridge- and H-bond-mediated contacts with all three domains of the CCT6 hemisphere subunits (Fig. 3e, f and Supplementary Table 4). The tubulin I domain also loosely interacted with CCT2 hemisphere subunits through association with the A-domain protrusion loop and C-termini of CCT7/5/2/4 (Fig. 3e). Indeed, tubulin showed an obviously larger interaction area with the CCT6 hemisphere subunits than with the CCT2 hemisphere ones (Supplementary Fig. 7l). Furthermore, inspection of the S3 structure also revealed the protrusion loop, loop^H10^, loop^H9^, C-terminus, and stem loop to be the main structural elements of TRiC involved in its interaction with tubulin (Fig. 3e, f and Supplementary Fig. 7m, n). Our TRiC-tubulin-S3 structure appeared overall comparable with the recent closed TRiC-tubulin structure^43^, with the dynamic tubulin I domain being slightly better resolved in our S3 map (Supplementary Fig. 6d, e).

In addition, our XL-MS data suggested that in the absence of added nucleotide, tubulin only cross-linked with the E domains of TRiC; while in the presence of ATP-AlFx, additional cross-links formed with the A/I domains of TRiC (Fig. 3g), indicating a shift of tubulin towards the A/I domains induced by ATP binding and hydrolysis. Indeed, more TRiC A and I domain regions were observed to be involved in the interaction with tubulin in the S2 and S3 states (Figs. 2j and 3e) than in the S1 state (Fig. 1h). Inspection of the S2 and S3 maps suggested that hydrolysis of ATP could induce considerable downward rotation of the A and E domains of TRiC from the open to closed state (Supplementary Fig. 7o). These substantial movements could dramatically reduce the chamber volume, and hence restrain the conformational landscape of the substrate and lead to a more stabilized substrate through intimate interactions with the TRiC A/I domains. In the meanwhile, the mechanical force generated from TRiC ring closure could provide extra energy to help the substrate overcome the energy barrier and transform towards its global energy minimum. This may be a general mechanism for TRiC-mediated substrate folding.

### Tubulin engages with closed TRiC through both electrostatic and hydrophilic interactions

**Fig. 4:**
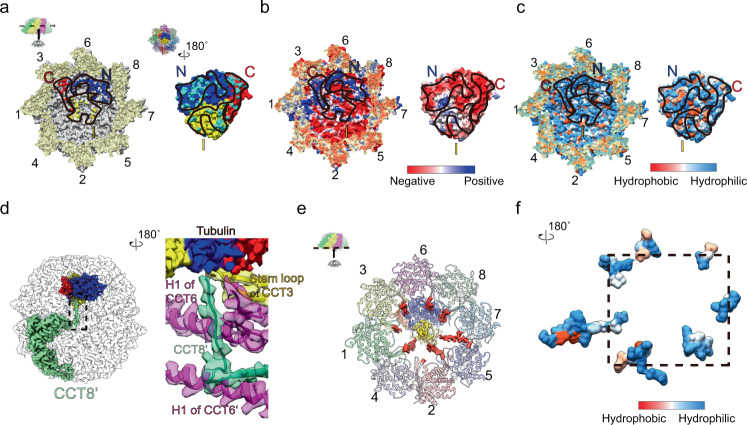
Engagement of tubulin with TRiC through electrostatic and hydrophilic interactions, and the roles of TRiC termini in tubulin folding in TRiC-tubulin-S3. **a** Interaction interface between TRiC and tubulin. The visualization direction and region are shown in the inset. The contact surface residues of TRiC within 4 Å distance from the N/C/I domains of tubulin are colored in blue, red, and yellow, respectively (left panel, black outlines indicate the tubulin footprint on the TRiC interior cavity wall). The contact surface residues of tubulin in proximity to TRiC are colored in cyan (right panel, black outlines indicate the TRiC footprint on the tubulin structure). **b** Electrostatic surface property analysis, suggesting complementary electrostatic interactions between CCT3/6/8 subunits of TRiC (mainly positively charged) and the N/C domains of tubulin (mostly negatively charged). **c** Hydrophilicity/hydrophobicity analysis, suggesting hydrophilic interactions between TRiC and tubulin. **d** Magnified view of the interaction network of the CCT8’ N-terminus. **e** Depiction showing that the resolved portions of the C-termini (red density) of all TRiC subunits except CCT4 were observed to form contacts with tubulin (central density). **f** Depiction of the hydrophilicity/hydrophobicity of the resolved C-termini of TRiC, showing these termini to be enriched in hydrophilic residues.

We then inspected the closed TRiC-tubulin-S3 structure to derive the properties of the interaction between tubulin and TRiC. Here the electrostatic surface inside the TRiC chamber was observed to be asymmetric, with the CCT6 hemisphere more positively charged and the CCT2 hemisphere relatively negatively charged (Fig. 4a, b), in line with previous reports on yeast and bovine TRiC^22^. Interestingly, the engaged tubulin N/C domains appeared negatively charged, and hence complementing the contacting CCT6 hemisphere (Fig. 4b). This observation was also consistent with our findings that numerous salt bridges formed between TRiC CCT6 hemisphere subunits and tubulin N/C domains (Fig. 3f). The electrostatic surface of the tubulin I domain was found to be not complementary with the related region of TRiC (Fig. 4a, b), consistent with the lack of a strong interaction between them (Fig. 3e and Supplementary Fig. 7l).

Inspection of the S3 structure also suggested an enrichment of hydrophilic residues at the interaction interfaces between TRiC and tubulin, creating a mostly polar TRiC-tubulin interface in the closed chamber (Fig. 4c). This type of polar interface was also seen in the TRiC-σ3 complex^52^. Taken together, our data suggested that, in general, the closed-state TRiC interacts with tubulin through a combination of electrostatic and hydrophilic interactions to stabilize this substrate and facilitate its folding.

We previously showed that yeast TRiC can close both rings in the presence of natural nucleotide ATP^17^. In the current work, our control experiment of cryo-EM analysis on human TRiC incubated for 30 s with 1 mM ATP, a physiological concentration of ATP in the cell^17,54^, further revealed the ability of human TRiC to close both rings, with a non-negligible population (27.2%) of such closed TRiC being detected (Supplementary Fig. 8a–c). This both-rings-closed TRiC map resembled that of the closed TRiC-tubulin-S3 (Supplementary Fig. 8d, e). Collectively, our data imply that the both-rings-closed conformation occurs in the TRiC conformational landscape driven by hydrolysis of natural nucleotide ATP. We therefore used nucleotide analog ATP-AlFx to trap the complex more in the closed state to facilitate a high-resolution structural study. Still, the ultimate judgment of whether the both-rings-closed TRiC exists in the crowded environment of the cell remains to be further explored through in situ cryo-ET studies of TRiC in normal cells.

### Cryo-EM map of TRiC-ADP

**Fig. 5:**
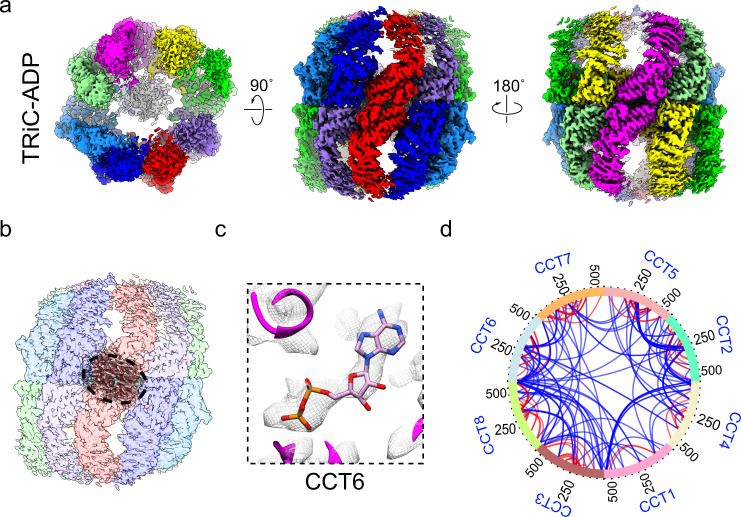
Cryo-EM and XL-MS analyses of TRiC in the presence of ADP. **a**, **b** Cryo-EM map of TRiC-ADP (**a**), showing the presence of the unstructured TRiC N-/C-tail density (in gray) but no substrate (**b**), suggesting that the substrate had been released. **c** Representative nucleotide density (in the ADP state) of CCT6 in TRiC-ADP. **d** XL-MS data showing no cross-links detected between TRiC and tubulin after the ADP incubation. Identified intra-subunit cross-links are shown in red, and inter-subunit cross-links in blue.

To describe the complete TRiC ATPase cycle, we produced a cryo-EM map of TRiC in the presence of ADP to a resolution of 3.3 Å (termed TRiC-ADP, Fig. 5a, b and Supplementary Figs. 1e, 9, and 10a, b). Inspection of this map indicated TRiC was in the open conformation, overall similar to that of TRiC-NPP (Supplementary Fig. 10c), but with all of the subunits loaded with ADP (Fig. 5c and Supplementary Fig. 10d). Noteworthy, there was no engaged tubulin density was now observed within the TRiC chamber, but only the central unstructured TRiC termini density remained (Fig. 5b). Corroborating this picture, our XL-MS data showed no cross-links between TRiC and tubulin after the ADP incubation (Fig. 5d)—indicating that, under our experimental conditions, loading of ADP to TRiC could contribute to a release of tubulin from TRiC chamber regardless of the tubulin folding status, and the remaining central density between the rings was indeed corresponding to the unstructured TRiC N-/C-termini. We performed further 3DVA on the dataset and found TRiC-ADP to be very dynamic, with all the subunits (including the most stable CCT6) displaying outward/inward tilting motions (Supplementary Fig. 10e and Supplementary Video 2). We then postulate that the dynamic nature of TRiC-ADP may contribute to the release of the substrate in this state. Still, we should mention that here we used the TRiC-ADP structure to mimic the ADP-binding state that, after ATP hydrolysis and γ-phosphate release, TRiC re-opened the rings and then released the substrate.

### The C- and N-termini of TRiC play distinct roles in substrate folding and TRiC allosteric coordination

Here, we observed an unstructured tail density centrally located between the two equators in each of the four open-state TRiC structures (TRiC-NPP, S1, S2, and TRiC-ADP, Figs. 1e, g,  2b, and Fig. 5b). Moreover, in both the S1 and S2 structures, the resolved portions of the TRiC C-termini and the extended unstructured tail apparently formed contacts with the tubulin substrate and hence potentially stabilized the substrate (Figs. 1g, h and  2b, j).

Strikingly, in the closed TRiC-tubulin-S3 structure, the N-terminus of almost all of the TRiC subunits were stabilized and very well resolved (Supplementary Fig. 11). Specifically, the N-termini of all subunits except CCT4 were observed to contact H1 of the neighboring subunit, and those of CCT2/4/5/7/8 were observed to be involved in the inter-ring allosteric network (Supplementary Figs. 11a–g). This observation was consistent with our previous finding in yeast TRiC, suggesting that the extra layer consisting of an N-terminal allosteric network could play important roles in TRiC ring closure^17^. Intriguingly, we observed the N-terminus of CCT8’ from the trans ring adopting a bent conformation and extending all the way to the cis-ring to associate with the tubulin N domain (Fig. 4d), in addition to being involved in the inter-ring allosteric network by linking the CCT6/6’ N-termini and CCT3 stem loop together. This observation indicated that CCT8 may play a critical role in substrate stabilization/folding besides being involved in TRiC inter-ring cooperativity.

Notably, in the closed S3 state, we also resolved a portion of the C-terminal extension for most TRiC subunits (except CCT4), and captured the uncharacterized direct interactions between these C-terminal extensions of TRiC and tubulin (Fig. 4e). These C-terminal extensions, mostly hydrophilic (Fig. 4f), appeared stretched out from the surrounding subunits towards the center of the TRiC chamber to form physical contacts with tubulin, and hence appeared like a net holding and stabilizing tubulin within the TRiC chamber (Fig. 4e), which may facilitate folding of tubulin. Consistent with this picture, the C-termini of GroEL was suggested to form contacts with its substrate, which could enhance and accelerate substrate folding, and the hydrophilic residues on the C-termini were suggested to be critical for substrate folding^55–58^.

## Discussion

**Fig. 6:**
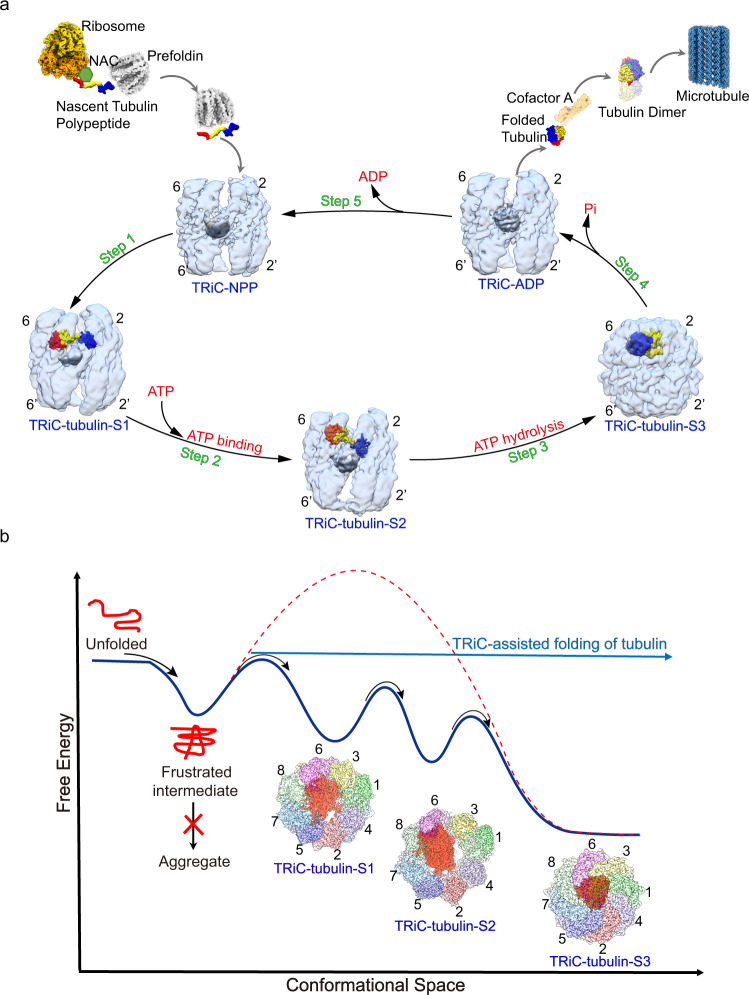
Proposed pathway and mechanism of TRiC-mediated tubulin folding. **a** Proposed pathway of tubulin folding mediated by TRiC associated with its ATP-driven conformational cycle. After being translated from ribosome, nascent tubulin polypeptides are delivered to TRiC-NPP by co-chaperone prefoldin. Tubulin is then released inside the TRiC chamber, and makes contacts with the E domains of all TRiC subunits and with the unstructured termini of TRiC, to form the TRiC-tubulin-S1 state (step 1). After binding of ATP to TRiC, tubulin can gradually translocate upwards to associate with the A/I domains of CCT6/8 and keep contacts with the E domains, forming the TRiC-tubulin-S2 state (step 2). Once ATP-hydrolysis triggers TRiC ring closure, forming the TRiC-tubulin-S3 state, the generated mechanical force together with the directional contacts and constraints on tubulin could facilitate tubulin folding towards its native state (step 3). Subsequently, in the γ-phosphate-released open TRiC-ADP state, the associated tubulin could be released from the TRiC chamber (step 4). The β-tubulin could be capped by cofactor A, and then assemble into tubulin heterodimers, with these heterodimers assembling into the microtubule. Finally, TRiC could then release ADP to revert to the NPP state (step 5). All TRiC maps were low-pass-filtered to 8 Å for easier visualization. TRiC is shown in transparent light blue, with its central unstructured N-/C-termini in non-transparent gray density and CCT2/6 labeled. **b** Hypothetical energy landscape of tubulin folding assisted by TRiC. Nascent tubulin in the absence of TRiC assistance is prone to form aggregates or needs to overcome a high-energy barrier to achieve its native state. Engagement of tubulin with TRiC in a manner associated with the TRiC ATPase cycle could potentially confine the energy landscape of tubulin and lower its energy barrier, resulting in stabilization of tubulin and, finally becoming folded into a substantially near-native state.

Tubulin is the building block of the microtubule, which is critical to many cellular processes, and the eukaryotic chaperonin TRiC is required for tubulin biogenesis. Here, we captured a more complete picture of tubulin folding pathway mediated by TRiC along its ATPase cycle (Fig. 6), by acquiring six cryo-EM structures of human TRiC, with three of them with endogenously engaged tubulin in different folding stages (Figs. 1, 2, and 5). In the open TRiC-tubulin-S1 and -S2 states, we detected an extra density corresponding to tubulin within the cis-ring chamber of TRiC (Figs. 1g and 2b), not seen in the recent study^43^. Furthermore, our TRiC-ADP map and the XL-MS data together revealed that the tubulin was released from TRiC chamber after ADP binding to TRiC, while the retained central density is the TRiC immanent N-/C-termini (Fig. 5b, d). Importantly, our TRiC-tubulin-S3 structure revealed a near-natively folded tubulin engaged with closed TRiC in one chamber, primarily with the A/I domains of the CCT3/6/8 subunits through electrostatic and hydrophilic interactions (Fig. 4a–c). We also showed that, in the closed S3 state, the N-terminus of CCT8 from the opposite ring and the C-terminal extensions of almost all TRiC subunits may play a role in the stabilization and folding of tubulin (Fig. 4d–f), not reported in the recent study^43^.

Here we propose a complete picture of the pathway and mechanism of TRiC-mediated tubulin folding accompanying the ATPase cycle of TRiC (Fig. 6a). After being translated from ribosomes, nascent tubulin polypeptides are delivered to TRiC by co-chaperon prefoldin to enter its folding pathway associated with the TRiC ATPase cycle^49,59^. Subsequently, tubulin can be released into TRiC chamber and located above the unstructured termini of TRiC, making contacts with the E domains of all the TRiC subunits, to form the TRiC-tubulin-S1 state, in which the tubulin is relatively dynamic (step 1). After binding of ATP to TRiC, with the resulting stabilization of the folding machinery, tubulin can be gradually translocated upward to associate with the A/I domains of CCT6/8 in addition to maintaining contacts with the E domains of all the subunits (step 2). Once hydrolysis of ATP triggers TRiC ring closure (with the involvement of TRiC N-termini), C-terminal extensions of TRiC and the N-terminus of CCT8 from the opposite ring could stabilize and potentially facilitate the upward translocation of tubulin to result in the formation of intimate electrostatic and hydrophilic interactions between the A domains of CCT6 hemisphere subunits and the N/C domains of tubulin. These directional contacts and constraints on the N/C domains of tubulin could facilitate its folding towards the native state, and the mechanical force generated by TRiC ring closure including the considerable downward rotation of the A domain of every TRiC subunit could be propagated to tubulin and drive it to overcome the energy barrier to transform towards the global minimum reaching the folded state (step 3). Subsequently, in the γ-phosphate-released open TRiC-ADP state, the associated tubulin could be released from the TRiC chamber (step 4). Based on previous reports^60–62^, the β-tubulin I domain could then be capped by cofactor A, followed by the assembly of tubulin heterodimers with the assistance of cofactors C, D, and E. Finally, the tubulin heterodimers assemble into the microtubule, which goes on to perform its biological functions.

Without TRiC-assisted folding, nascent tubulins are prone to form aggregates due to the high-energy barrier to any spontaneous folding reaching the native folded state^63^. In the current work, by capturing multiple intermediate states of tubulin folding assisted by TRiC, we were able to derive a hypothetical energy landscape of the three distinct states of TRiC-tubulin on the basis of nucleotide binding status, complex stability, and population distribution (Fig. 6b). In TRiC-tubulin-S1, nascent tubulin could potentially become stabilized by its engagement with TRiC, leading it to overcome the first energy barrier to reach a lower level in the energy landscape. Still, the tubulin at this stage remains quite dynamic, accompanying the dynamic nature of TRiC in the NPP state with only CCT3/6/8 subunits preloaded with ADP (Supplementary Fig. 3f, g). The subsequent binding of ATP to all the TRiC subunits could drive TRiC and associated tubulin to overcome the next barrier and to stabilize in the steadier TRiC-tubulin-S2 state, evidenced by the observation of the engaged tubulin density here becoming further stabilized and appearing larger. Only when hydrolysis of ATP provides sufficient chemical energy to enable TRiC to overcome the final energy barrier, shutting both rings and becoming transformed to the most stable TRiC-tubulin-S3 state, does the confined tubulin now fold into its near-native state inside the closed TRiC chamber. This step may be the rate-limiting step in the tubulin folding process. Thus, the ATP-mediated gradual stabilization of TRiC guides tubulin along a pathway that avoids deep kinetic traps in the folding energy landscape. That is to say, binding of tubulin to TRiC could lower the folding energy barrier of tubulin and result in its stabilization, so that it eventually reaches its folded state accompanying ATP-driven TRiC ring closure.

TRiC subunits have been previously demonstrated to display a gradient of ATP affinities^28^, as well as differences in nucleotide binding^16,20,29^ and consumption^17^, with these features closely related to the structural asymmetry among TRiC subunits^20,25,64^. In the current study, our results further showed that, in the TRiC-NPP state, the ADP bound to CCT3/6/8 may play a role in stabilizing these subunits, making them appear less intrinsically dynamic than the other subunits (Fig. 1d, f). CCT3/6/8 may thus serve as a dock for an initial engagement of tubulin with TRiC (Figs. 1h and  2j), and eventually form close electrostatic and hydrophilic contacts with the N/C domains of tubulin in its nearly folded state (Figs. 3f and  4a–c). Consistently, previous studies also suggested important roles of CCT3/6/8 in recognition of other substrates such as mLST8, reovirus σ3 capsid protein, and AML1-175^26–28^. Taken together, the asymmetry of TRiC subunits in structural features and nucleotide consumption may contribute to TRiC-assisted substrate folding.

In addition, we found that along with the folding of substrate from a relatively disordered state to an ordered state, TRiC also appears to follow a similar trend: transforming from the relatively dynamic and asymmetric open S1 state to the stabilized, rather symmetrical both-rings-closed S3 state. And during this process, the nucleotide occupancy status also appears to change, from an initial partial occupancy in the NPP state to the full occupancy in all the subunits of TRiC, with the nucleotide states becoming more homologous (Supplementary Figs. 3f and 7g, h). In the nucleotide binding and consumption, as well as ring closure process, the asymmetric machinery—TRiC operates using a stepwise mechanism^17,20^ rather than a concerted manner as in the Group I system^65,66^. As has been suggested previously^67^, the asymmetry or non-concerted nature of TRiC in the open state could be beneficial for its ability to recognize and engage with diverse substrates. Moreover, despite our study and most other studies having revealed only one substrate engaged with TRiC^8,12,29,30,52,68^, a previous crystal structure showed one TRiC bound to two tubulins, one per ring^16^, implying that two substrates could interact with a TRiC complex simultaneously at least under certain physiological conditions^67^ and that both rings of TRiC may have the ability to perform substrate folding concurrently for efficient protein quality control under these conditions.

More recently, a cryo-EM study on recombinant prefoldin/TRiC/tubulin system was published^69^, in which an open-state TRiC bound with prefoldin and four closed-state TRiC containing β-tubulin at different folding status were presented. We would like to point out that in our open-state TRiC-tubulin-S1 and -S2 maps, we found and assigned an extra density in the cis-ring chamber of TRiC as tubulin, while this density was not seen in the open TRiC structures from the abovementioned one^69^ and the recent report^44^. Instead, they assigned the central density between the TRiC rings as substrate. However, this central density has been observed in all our four open-state TRiC maps (including the free NPP-TRiC, TRiC-tubulin-S1 and TRiC-tubulin-S2, as well as TRiC-ADP), and was assigned as N-/C-termini of TRiC. Indeed, this central density was also observed in previous bovine and human free TRiC open-state maps^8,49^ (Extended Data Fig. 4f, g). Related to this, our TRiC-ADP map showed that other than the remaining central density, the tubulin density has been released from TRiC chamber after ADP incubation (Fig. 5b), in line with our XL-MS data (Fig. 5d), further suggesting that the retained central density is the TRiC immanent N-/C-termini. Still, we cannot rule out the possibility that this difference might be due to divergent in sample preparation strategy (recombinant vs. endogenous purification, with vs. without PFD), and data processing strategy between their and our studies.

In summary, our study provides a thorough picture of the pathway and conformational landscape of TRiC-mediated tubulin folding accompanying the ATPase cycle of the folding machinery. Furthermore, our determination of the interaction sites between tubulin and the closed TRiC chamber could be beneficial for the development of novel and effective therapeutic agents specifically targeting TRiC-tubulin interactions.

## Methods

### Purification of human TRiC

Human TRiC was purified from HEK293F cells according to the published protocol but with some modifications^29,49,70^. Briefly, the pellet was lysed with iced MQA-10% glycerol buffer (50 mM NaCl, 20 mM HEPES pH 7.4, 5 mM MgCl_2_, 1 mM DTT, 10% glycerol, 1 mM PMSF, and 2 mM ATP) using one Protease Inhibitor Cocktail Tablet (Roche) per 100 mL of the lysate. The lysed material was then subjected to centrifugation (at 20,000 × *g* for 30 min to remove cellular debris and nuclei and then at 140,000×*g* for 1.5 h to remove the ribosome) in order to separate the cytoplasmic fraction. The resulting supernatant was filtered using a 0.44 μm filter membrane and then passed through a Q Sepharose column (GE Healthcare). TRiC was eluted in a gradient from 40 to 80% MQB-5% glycerol (MQA with 1 M NaCl). The fractions containing TRiC were pooled, diluted with MQA to ensure an NaCl concentration of about 100 mM, and applied to a HiTrap Heparin HP column (GE Healthcare). TRiC was here eluted in a gradient from 20 to 65% MQB-5% glycerol. The fractions containing TRiC were pooled and incubated with 10 mM ATP on a shaker (220 rpm, 37 °C, 30 min) to allow TRiC to cycle and release substrate before going through gel filtration chromatography (GFC). The sample was then concentrated down to 0.5 mL and loaded onto a Superose 6 Increase 10/300 GL column (GE Healthcare) with MQA-5% glycerol without ATP. TRiC eluted at about 13.0–15.5 mL of the size-exclusion column, consistent with that of a 1-MDa complex. Finally, the resulting TRiC-containing eluate was subjected multiple times to buffer exchange to remove the remaining ATP in the buffer—and, in this way, we obtained biologically active TRiC (Supplementary Fig. 1a, b).

Note that TRiC-mediated folding of tubulin is associated with release of predominantly nonnative forms of tubulin from the chaperonin, and with the majority of released tubulin requiring further rounds of binding/release to reach its native state^71^. In our experimental conditions, it was possible that the tubulin in TRiC-tubulin-S1 had already gone through one or several ATPase cycles.

### ATPase activity assay

The ATP-hydrolysis rate of TRiC was measured by performing an NADH-coupled assay^72^. In general, in this assay, each ATP-hydrolysis event allows for a pyruvate kinase (PK)-catalyzed conversion of one molecule of phosphoenolpyruvate into pyruvate, with pyruvate then converted to lactate by l-lactate dehydrogenase, resulting in oxidation of a single NADH molecule. Loss of NADH over time, a measure quantifiably proportional to the ATP-hydrolysis rate, was monitored in the current work by measuring the decrease in absorbance of light at a wavelength of 340 nm. All the assays were conducted at room temperature in a buffer containing 10 mM HEPES/NaOH pH 7.5, 50 mM NaCl, and 10 mM MgCl_2_, in the presence of 1 mM ATP. Experiments were performed in triplicate using 0.3 μM of the protein complex. Absorbance was measured in a reaction volume of 200 µl using a 96-well plate reader. Data analysis was performed using GraphPad Prism 8.

### ADP/ATP ratio assay

To identify the form of the nucleotide in our purified human TRiC, we carried out luciferin-luciferase reactions with an ADP/ATP ratio assay kit^73^ (Sigma-Aldrich). To release the bound nucleotide from TRiC for the measurement, the TRiC sample was first digested with proteinase K, according to a previously published protocol^74^ with minor modifications. The TRiC proteolytic-digestion experiments were carried out by adding a 1 µl aliquot of 2 mg/ml proteinase K to a 19 µl aliquot of 4.5 mg/ml TRiC in dilution buffer (20 mM HEPES-KOH pH 7.4, 50 mM NaCl, 5 mM MgCl_2_, 1 mM DTT, and 5% glycerol). The digestion was performed at 37 °C for 1 h. The reactions were then terminated by adding PMSF (to a final concentration of 5 mM) into the reaction mixture and waiting for 10 min. Afterward, the nucleotide form in the TRiC sample was identified by using the ADP/ATP ratio assay kit according to the manufacturer’s protocol, and the RLU values of ATP and ADP were measured with a Synergy Neo2 multimode reader (BioTek).

### Cross-linking and mass spectrometry analysis

The purified TRiC was cross-linked by reacting it with bis[sulfosuccinimidyl] suberate (BS^3^) (Sigma), with a spacer arm of 11.4 Å between cross-linked Cα carbons, on ice for 2 h. The final concentration of the crosslinker was 2 mM. The reaction was then terminated by using 50 mM Tris-HCl pH 7.5 at room temperature for 15 min. For the sample containing ATP-AlFx, the purified TRiC was incubated with 1 mM ATP, 5 mM MgCl_2_, 5 mM Al(NO_3_)_3_, and 30 mM NaF for 1 h at 37 °C, and the resulting product was cross-linked by using BS^3^ following the abovementioned protocol. Cross-linked complexes were precipitated and digested for 16 h at 37 °C by subjecting them to trypsin at an enzyme-to-substrate ratio of 1:50 (w/w). The tryptic-digested peptides were desalted and loaded on an in-house-packed capillary reverse-phase C18 column (length of 40 cm, 100 µm ID × 360 µm OD, 1.9-µm particle size, pore diameter of 120 Å) connected to an Easy LC 1200 system. The samples were analyzed with a 120 min-HPLC gradient of 6% to 35% of buffer B (buffer A: 0.1% formic acid in water; buffer B: 0.1% formic acid in 80% acetonitrile) at 300 nL/min. The eluted peptides were ionized and directly introduced into a Q-Exactive mass spectrometer using a nano-spray source. Survey full-scan MS spectra (from *m/z* 300 to 1800) were acquired using an Orbitrap analyzer with a resolution *r* = 70,000 at an *m/z* of 400. Cross-linked peptides were identified and evaluated using pLink2 software^75^.

### Cryo-EM sample preparation

To prepare a vitrified sample of TRiC, the purified TRiC was diluted to 1.2 mg/ml, and an aliquot of 2 μl of this sample was applied onto a plasma-cleaned holey carbon grid (Quantifoil, R1.2/1.3, 200 mesh). The grid was blotted with Vitrobot Mark IV (Thermo Fisher Scientific) and then plunged into liquid ethane cooled by liquid nitrogen. To prepare the sample of TRiC in the presence of 1 mM ATP-AlFx, the purified TRiC was diluted to 1.2 mg/ml, and incubated with 1 mM ATP, 5 mM MgCl_2_, 5 mM Al (NO_3_)_3_, and 30 mM NaF at 37 °C for 1 h prior to freezing. To prepare the sample of TRiC with 1 mM ADP, the purified TRiC was diluted to 1.2 mg/ml, and incubated with 1 mM ADP and 5 mM MgCl_2_ at 37 °C for 1 h prior to freezing. To prepare the sample of TRiC in the presence of 1 mM ATP, the purified TRiC was diluted to 1.2 mg/ml, and incubated with 1 mM ATP and 5 mM MgCl_2_ at 37 °C for 30 s before freezing. We then followed the abovementioned procedure to prepare the vitrified sample in each case.

### Data acquisition

For each of the experimental sample conditions mentioned above, except for the TRiC-ATP sample, cryo-EM movies of the sample were collected using a Titan Krios electron microscope (Thermo Fisher Scientific) operated at an accelerating voltage of 300 kV with a nominal magnification of 18,000x (yielding a pixel size of 1.318 Å, Table 1). The movies were recorded on a K2 Summit direct electron detector (Gatan) operated in super-resolution mode under a low-dose condition in an automatic manner using SerialEM^76^. The exposure time for each frame was 0.2 s, and the total accumulation time was 7.6 s, leading to a total accumulated dose of 38 e^–^/Å^2^ on the specimen. For the TRiC-ATP sample, the movies were collected at a magnification of 81,000x (yielding a pixel size of 0.89 Å, Table 1) utilizing a K3 direct electron detector (Gatan) operated in the counting mode under a low-dose condition in an automatic manner using EPU (Thermo Fisher Scientific). Each frame was exposed for 0.05 s, and the total accumulation time was 2 s, leading to a total accumulated dose of 50 e^–^/Å^2^ on the specimen.

**Table. 1:** Cryo-EM data collection, processing, and model validation statistics.

	TRiC-NPP (EMDB-32922) (PDB- 7X0A)	TRiC-tubulin-S1 (EMDB-32903) (PDB- 7WZ3)	TRiC-tubulin-S2 (EMDB-32989) (PDB- 7X3J)	TRiC-tubulin-S3 (EMDB-32923) (PDB- 7X0S)	TRiC-closed (EMDB-32926) (PDB- 7X0V)	TRiC-ADP (EMDB-32993) (PDB- 7X3U)	TRiC-ATP-closed (EMDB-33025) (PDB- 7X6Q)	TRiC-ATP-open (EMDB-33053) (PDB- 7X7Y)
*Data collection and processing*								
Magnification	18,000						81,000	
Voltage (kV)	300						300	
Detector	K2 summit						K3	
Electron exposure (e^–^/Å^2^)	38						50	
Defocus range (μm)	–0.8 to –2.5						–0.8 to –2.5	
Pixel size (Å)	1.318						0.89	
Symmetry imposed	C1						C1	
Initial particle images (no.)	761,665	761,665	943,101	943,101	943,101	884,769	351,262	351,262
Final particle images (no.)	185,216	115,666	87,764	103,406	62,412	239,315	41,646	111,413
Map resolution (Å)	3.1	4.1	4.2	3.1	3.2	3.3	4.5	3.8
FSC threshold	0.143							
Map resolution range (Å)	3.0–8.0	3.8–8.4	3.8–8.4	2.8–4.5	2.9–4.5	3.0–8.0	3.5–5.5	3.4–6.4
*Refinement*								
Initial model used (PDB code)	5GW4; 5GW5	5GW4; 5GW5	5GW4; 5GW5	6KS6; 5JCO	6KS6	5GW4; 5GW5	6KS6	5GW4; 5GW5
Model resolution (Å)	4.2	7.4	7.2	3.4	3.6	3.6	4.5	4.2
FSC threshold	0.5							
Map sharpening *B* factor (Å^2^)	–76.7	–133.7	–144.4	–53.3	–80.4	–96.2	–135.8	–111.4
Model composition								
Non-hydrogen atoms	63,694	58,818	63,464	63,367	65,020	63,964	64,486	63,532
Protein residues	8314	7686	8241	8866	8440	8314	8440	8314
Ligands	6	6	16	48	48	16		
*B* factors (Å^2^)								
Protein	120.55	127.93	31.20	55.75	79.70	33.58	55.28	179.95
Ligand	57.86	45.81	11.53	51.16	72.45	8.06		
R.m.s. deviations								
Bond lengths (Å)	0.005	0.004	0.005	0.003	0.004	0.003	0.091	0.003
Bond angles (°)	0.533	0.996	1.105	0.665	1.072	0.655	1.669	0.886
Validation								
MolProbity score	1.39	1.13	1.40	1.43	1.54	1.17	1.35	1.44
Clashscore	3.67	2.17	4.21	3.19	4.23	1.78	3.45	5.18
Poor rotamers (%)	0.04	0.00	0.07	0.04	0.04	0.07	0.01	0.00
Ramachandran plot								
Favored (%)	96.50	97.25	96.82	95.36	95.10	96.48	96.68	97.09
Allowed (%)	3.47	2.75	3.17	4.64	4.90	3.43	3.16	2.91
Disallowed (%)	0.03	0.00	0.01	0.00	0.00	0.09	0.15	0.00

### Image processing and 3D reconstruction

We performed single-particle analysis mainly using Relion 3.1^77,78^ unless otherwise specified (Table 1). All images were aligned and summed using MotionCor2^79^. After CTF parameter determination using CTFFIND4^80^, particle auto-picking, manual particle checking, and reference-free 2D classification, particles with TRiC features were kept for further processing.

For the TRiC sample before the addition of nucleotides, after subjecting it to 2D classification, 709,192 particles remained (Supplementary Fig. 2a). These particles were subjected to 3D refinement and then were re-extracted and re-centered using the refinement coordinates. After one round of no-align 3D classification and another round of 3D classification, we then combined the substrate-containing particles (including class 1 from the first round and class 3 from the second round) together and performed another round of 3D classification, generating a substrate-containing class with 169,098 particles. After carrying out CTF refinement and polishing on these particles, we obtained a map with weak substrate density to a resolution of 4.0 Å. We subtracted the substrate density and performed a no-align 3D classification, and a good class with 68.4% of the particles were reverted to the original particles. We then performed a local refinement on these particles and reconstructed a 4.1-Å-resolution TRiC-tubulin-S1 map displaying better substrate density. For the substrate-free class 4 particles from the second round of 3D classification, we performed another round of 3D classification, resulting in a better class having 185,216 particles. After CTF refinement and Bayesian polishing, these particles were refined to produce a 3.1-Å-resolution TRiC-NPP map. The resolution estimation was based on the gold-standard Fourier shell correlation (FSC) criterion of 0.143.

For the dataset of TRiC in the presence of ATP-AlFx, after subjecting it to 2D classification, 544,809 particles remained (Supplementary Fig. 5a). These particles were subjected to 3D classification. The particles from class 4, displaying closed-state features, were subjected to another round of 3D classification, and a resulting better class with 168,472 particles was further refined to produce a 2.9-Å-resolution map with weak substrate density inside the TRiC chamber. Thus, we focused on the extra density inside the chamber and performed a no-align 3D classification, generating 4 classes. Particles from classes 1 and 2, appearing to have tubulin density, were combined (total of 103,406 particles) and refined to produce a 3.1-Å-resolution TRiC-tubulin-S3 map; class 3 (62,412 particles), having an extra tail density, were used to reconstruct a 3.2-Å-resolution TRiC-closed map. In addition, particles of classes 1–3 from the first round of 3D classification were subjected to another round of 3D classification and yielded a better class with 98,046 particles, which were refined to produce a 4.3-Å-resolution map. Through focused refinement and focused classification, we eventually obtained a 4.2-Å-resolution map with tubulin density in one ring, termed TRiC-tubulin-S2.

For the TRiC-ADP dataset, after subjecting it to 2D classification, 344,486 and 319,306 particles remained for datasets 1 and 2, respectively (Supplementary Fig. 9a). After subjecting them to refinement and re-centering, the particles were further cleaned using cryoSPARC through 2D classification and heterogeneous refinement. Then the good particles were subjected to two rounds of 3D classification in Relion 3.1. Subsequently, the better-resolved classes 3/4 from dataset 1 and classes 2/3 from dataset 2 were combined and subjected to one more round of 3D classification. The 239,315 particles of the better class were further refined to produce a 3.3-Å-resolution TRiC-ADP map.

For the TRiC-ATP dataset, after 2D classification, 225,085 particles remained (Supplementary Fig. 8a). These particles were refined and re-extracted, and further cleaned up using cryoSPARC by subjecting them to 2D classification and heterogeneous refinement to yield three classes. Class 1 open-state particles were subjected to another round of heterogeneous refinement, and the 111,413 particles of the resulting class 1 were refined to produce a 3.8-Å-resolution TRiC-ATP-open map. Closed-state particles from class 2 of the first round of heterogeneous refinement were subjected to another round of heterogeneous refinement, and particles from the class 2 were refined in Relion 3.1 to produce a 4.5-Å-resolution TRiC-ATP-closed map.

### Model building by performing flexible fitting

We built the homology models for human TRiC in the open and closed states and for tubulin (TUBB5) using the SWISS-MODEL server^81^—with, respectively, the cryo-EM structures of yeast TRiC in the open and closed conformations (PDB ID: 5GW4, 5GW5, 6KS6^17,20^) and the cryo-EM structure of human TUBB3 tubulin (PDB ID: 5JCO^82^) as templates. Afterwards, we refined each model against its corresponding cryo-EM map using Rossetta^83^, and then Phenix^84^. Furthermore, to improve the fitting between model and map, we performed real-space refinement using COOT^85^. Finally, we used Phenix again for the last round of flexible fitting of the entire complex.

We used UCSF Chimera and ChimeraX for generating figures and performing electrostatic surface property calculations^86,87^. Interaction surface analysis was conducted by using the PISA server^88^.

### Statistics and reproducibility

Statistical analyses were performed using GraphPad Prism 8.4.3. For all quantifications, data were plotted as mean ± SD for three independent replicates and dots of individual sample data are also presented. Comparisons between groups were performed using unpaired *t* test. *P* < 0.0001 was considered to indicate significance.

### Reporting summary

Further information on research design is available in the Nature Portfolio Reporting Summary linked to this article.

## Supplementary information

Peer Review File

Supplementary Information

Description of Additional Supplementary Files

Supplementary Video 1

Supplementary Video 2

Reporting Summary

## Data Availability

All data presented in this study are available within the figures and in the Supplementary Information. Uncropped scans are provided in Supplementary Fig. 12. Cryo-EM maps and the associated models have been deposited in the EMDB and Protein Data Bank, respectively, with the accession IDs as follows: TRiC-NPP (EMDB-32922, PDB-7X0A), TRiC-tubulin-S1 (EMDB-32903, PDB-7WZ3), TRiC-tubulin-S2 (EMDB-32989, PDB-7X3J), TRiC-tubulin-S3 (EMDB-32923, PDB-7X0S), TRiC-closed (EMDB-32926, PDB-7X0V), TRiC-ADP (EMDB-32993, PDB-7X3U), TRiC-ATP-closed (EMDB-33025, PDB-7X6Q), TRiC-ATP-open (EMDB-33053, PDB-7X7Y).
