# Supplementary material for: Pathway and mechanism of tubulin folding mediated by TRiC/CCT along its ATPase cycle revealed using cryo-EM
Source: Commun Biol. 2023 May 16;6:531. doi: 10.1038/s42003-023-04915-x (PMC10188570; doi:10.1038/s42003-023-04915-x)
Supplement: Supplementary file 2 — Supplementary Information [file 42003_2023_4915_MOESM2_ESM.pdf]

Supplementary Information

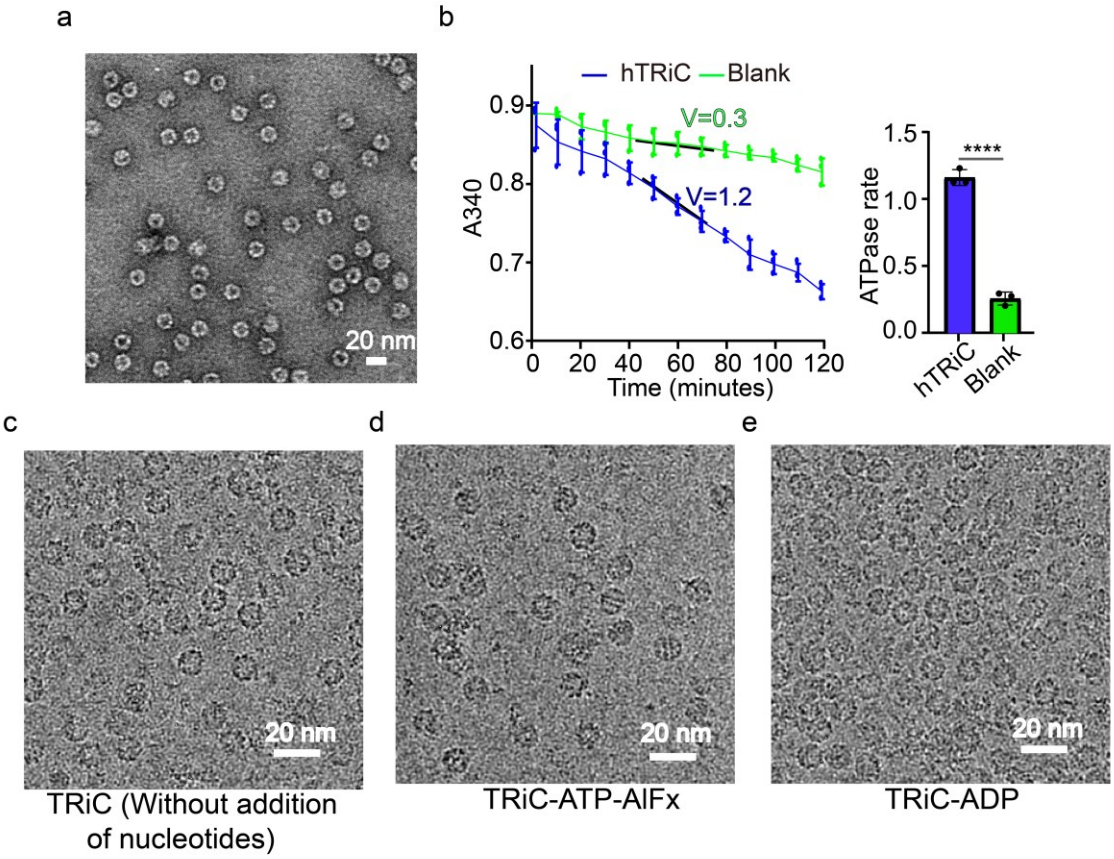

Supplementary Figure 1 | Human TRiC purified from HEK293F cells. a, Representative negative-stain EM image of purified TRiC. **b**, NADH-coupled enzymatic assays of our purified TRiC and a blank (TRiC buffer without proteins). The results revealed that TRiC was biologically active and could hydrolyze ATP. The ATPase rates (V, with unit of "mole ATP/ [mole TRiC • min]") determined by fitting the linear part of the ATP hydrolysis reaction curves are also provided. An analysis of the significance of the difference between the results (right) suggested that the TRiC sample showed a significantly higher ATPase activity than did the blank, with a statistical significance of ****$P < 0.0001$. For all quantifications, data were plotted as mean ± SD for three independent replicates. **c–e**, Representative cryo-EM images of TRiC sample before adding nucleotides (**c**), in the presence of ATP-AlFx (**d**) or ADP (**e**).

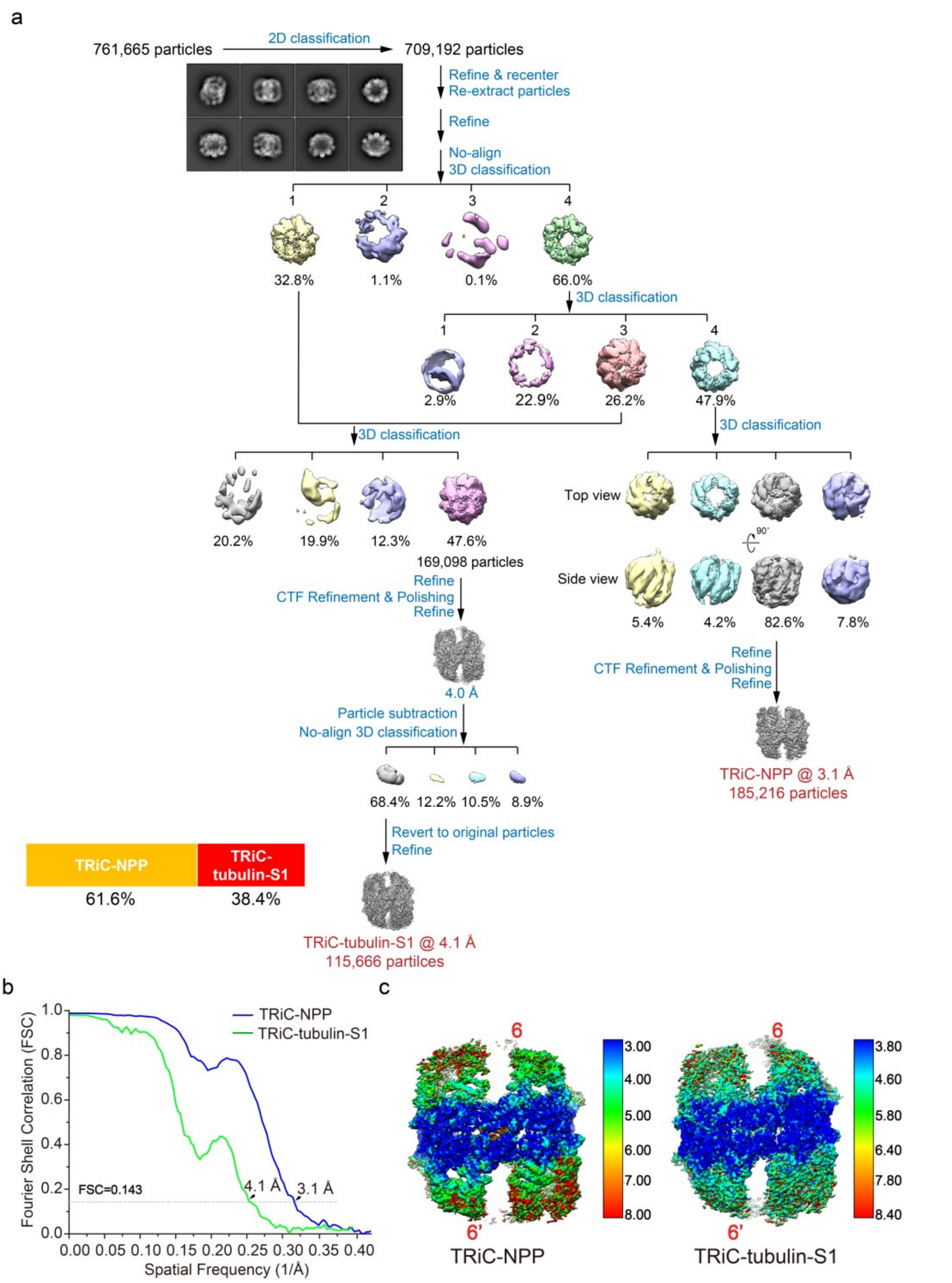

Supplementary Figure 2 | Cryo-EM analysis on the endogenously purified TRiC. **a**, Procedure used to process the TRiC cryo-EM data. The reference-free 2D class averages and population distributions of TRiC-NPP and TRiC-tubulin-S1 are also presented. **b–c**, Resolution estimations for TRiC-NPP and TRiC-tubulin-S1 according to the gold-standard FSC criterion of 0.143 (**b**), and local resolution evaluations (**c**).

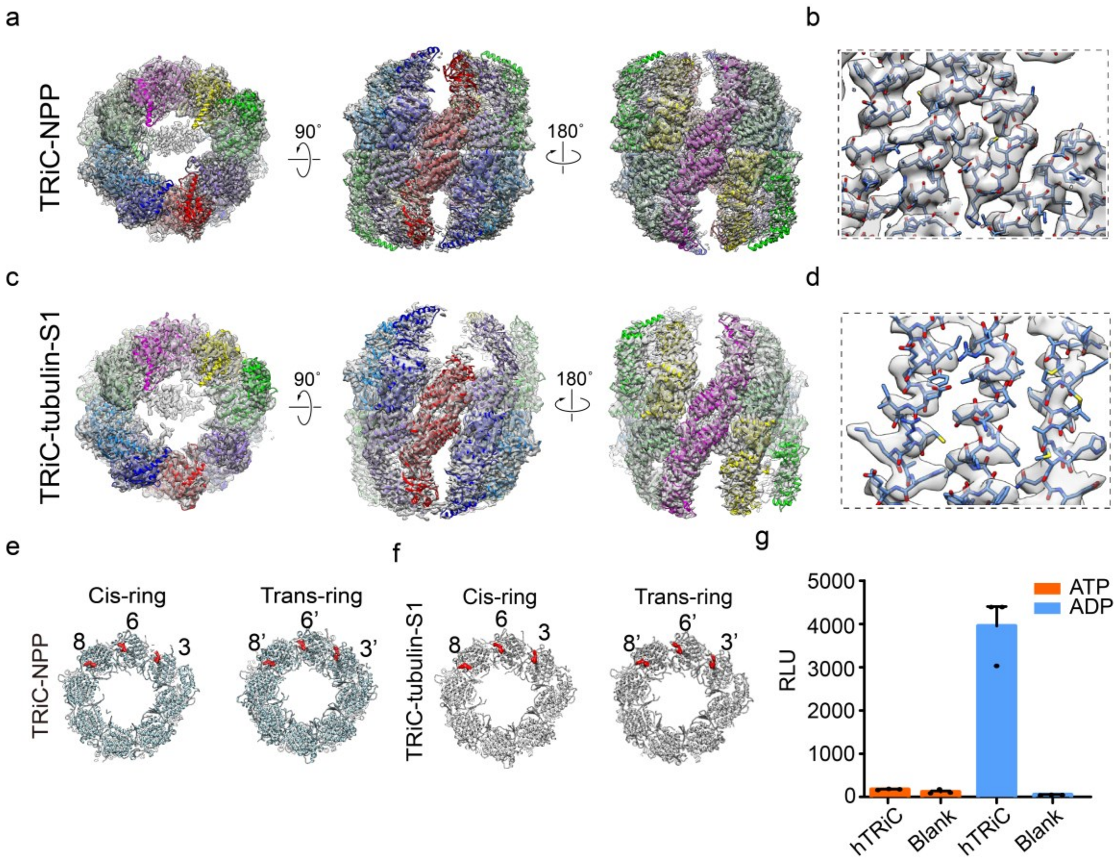

Supplementary Figure 3 | Atomic models of TRiC-NPP and TRiC-tubulin-S1. **a,c,** Atomic models of TRiC-NPP (**a**) and TRiC-tubulin-S1 (**c**) match well with the corresponding cryo-EM maps. **b,d,** Representative high-resolution structural features of TRiC-NPP (**b**) and TRiC-tubulin-S1 (**d**). **e–f**, Nucleotide occupancy statuses of TRiC-NPP (**e**) and TRiC-tubulin-S1 (**f**), with CCT3/6/8 from both rings having nucleotide density (in red) in their nucleotide pockets. **g**, ATP/ADP ratio analysis of TRiC. The relative light unit (RLU) values were measured for the sample with TRiC buffer as a blank. For all quantifications, data were plotted as mean ± SD for three independent replicates.

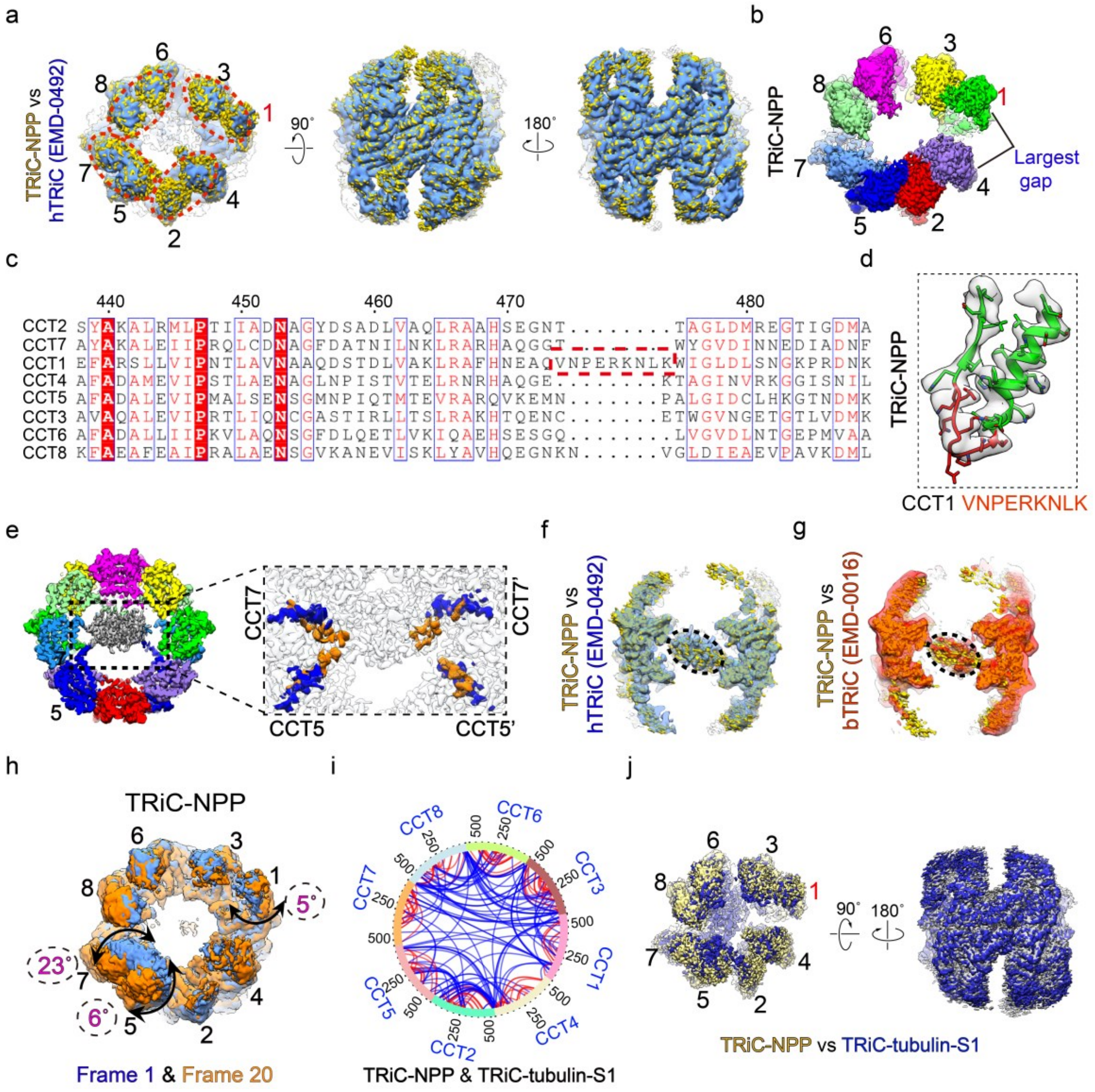

Supplementary Figure 4 | TRiC-NPP and TRiC-tubulin-S1 conformations and subunit configurations. **a**, Overlay of our TRiC-NPP map (yellow) and the reported apo hTRiC map (cornflower blue, EMDB: 0492), indicating no obvious conformational differences between the two structures. The tetramer-of-dimers pattern is indicated by dotted red circles. **b**, Structure of TRiC showing CCT1 and CCT4 displaying the largest gap between their A domains. **c–d**, Sequence alignment of the eight subunits of hTRiC, revealing a unique insertion (indicated by red dashed frames) in the E domain of CCT1 (**c**), and the structure of this insertion in TRiC-NPP (**d**). **e**, Depiction of the TRiC-NPP map showing the central tail density symmetrically contacting the N-/C-termini of CCT5/7 and CCT5'/7' from both rings. In the magnified cut-off top view (right), only the resolved N-/C-termini of CCT5/5' and CCT7/7' are colored, with the C-terminal

tails in blue and N-terminal tails in orange. **f–g**, Central slice view of TRiC-NPP (yellow) overlaid with apo hTRiC (cornflower blue, EMDB: 0492[50]) (**f**), and with apo bovine TRiC (red, EMDB: 0016) (**g**), to show their similar tail density features (indicated by black dashed ellipsoid). **h**, Results for the 3DVA of the TRiC-NPP dataset. This analysis suggested that CCT7/5/1 underwent continuous outward/inward tilting motions of up to ~23°/6°/5°, respectively. Frame 1 (cornflower blue) and frame 20 (orange), together showing the maximum extent of the motion, are displayed. **i**, XL-MS-derived circular plot of all cross-links of the TRiC portion, with intra-subunit cross-links shown in red, and inter-subunit cross-links in blue. The detected cross-links within the TRiC complex fulfill the spatial geometry constraints of the linked amino acids, validating the reliability of our XL-MS data. **j**, TRiC-NPP map (yellow) overlaid with the TRiC-tubulin-S1 map (medium blue), indicating no obvious conformational differences between them.

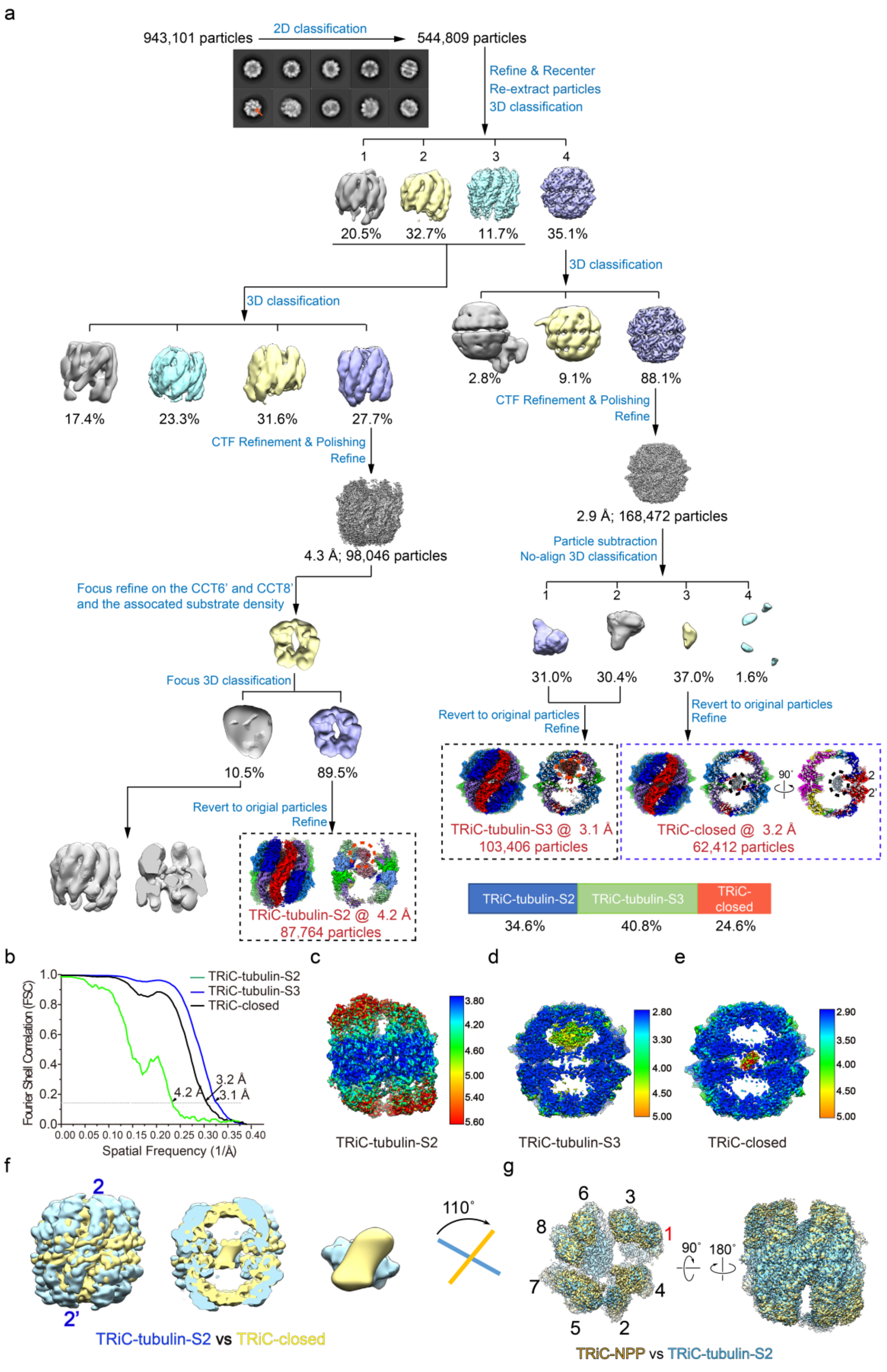

Supplementary Figure 5 | Cryo-EM analysis on the data of TRiC in the presence

of ATP-AlFx. a, Procedure used to process the cryo-EM data of TRiC in the presence of ATP-AlFx. The reference-free 2D class averages and the population distributions of the three obtained states are also presented. Besides, the central slice view shown of the TRiC-closed map revealed the tail density attached to the CCT2/2' subunit pair. **b**, Resolution estimations of the TRiC-tubulin-S2, -S3, and TRiC-closed maps according to the gold-standard FSC criterion of 0.143. **c–e**, Local resolution evaluations for the S2 (**c**), S3 (**d**), and TRiC-closed (**e**) maps. **f**, Overlay of the TRiC-tubulin-S2 and TRiC-closed maps. When visualized from the CCT2 side, the unstructured tail of TRiC-closed rotated clockwise by about 110° relative to that of TRiC-tubulin-S2. The two maps were low-pass-filtered to 8 Å for better visualization of the tail density. **g**, Overlay of the TRiC-NPP (yellow) and -S2 maps (sky blue), indicating the overall similar conformations of TRiC.

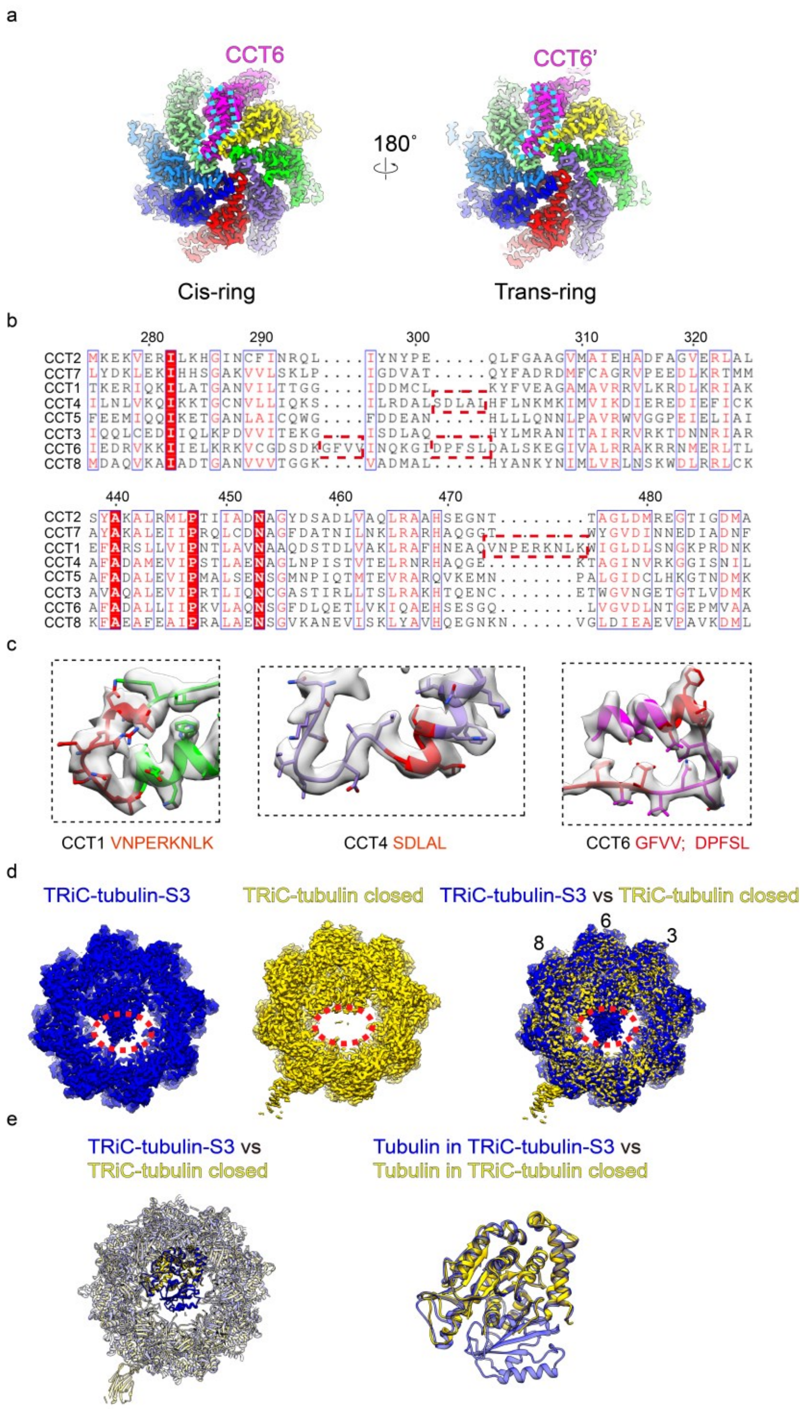

Supplementary Figure 6 | Features facilitating identification of the TRiC subunits in the TRiC-tubulin-S3 map and structural comparison between the TRiC-tublin-S3 and the recent TRiC-tubulin closed state maps. a, Depictions of the S3 map in which the unique kink feature (enclosed in a dotted blue line) in the apical protrusion H8 of CCT6 was unambiguously visualized in both rings, substantiating the on-axis location of CCT6. **b**, Sequence alignment of the eight subunits of hTRiC, revealing four unique insertions (indicated by red dashed frames) in CCT1/4/6. **c**, Related structures

of the four insertions (in red), all resolved in our S3 map. **d**, Top view of the TRiC-tubulin-S3 and TRiC-tubulin closed state maps (EMDB: 12607) [44], and an overlay of the two maps. TRiC A domains were omitted for visualization of the bound tubulin density. **e,** Overlay of the TRiC-tubulin-S3 and TRiC-tubulin closed state structures, revealing similar overall orientations and binding locations of tubulin within the respective TRiC chambers of the two structures, but a slightly better resolved tubulin I domain in our TRiC-tublin-S3 structure.

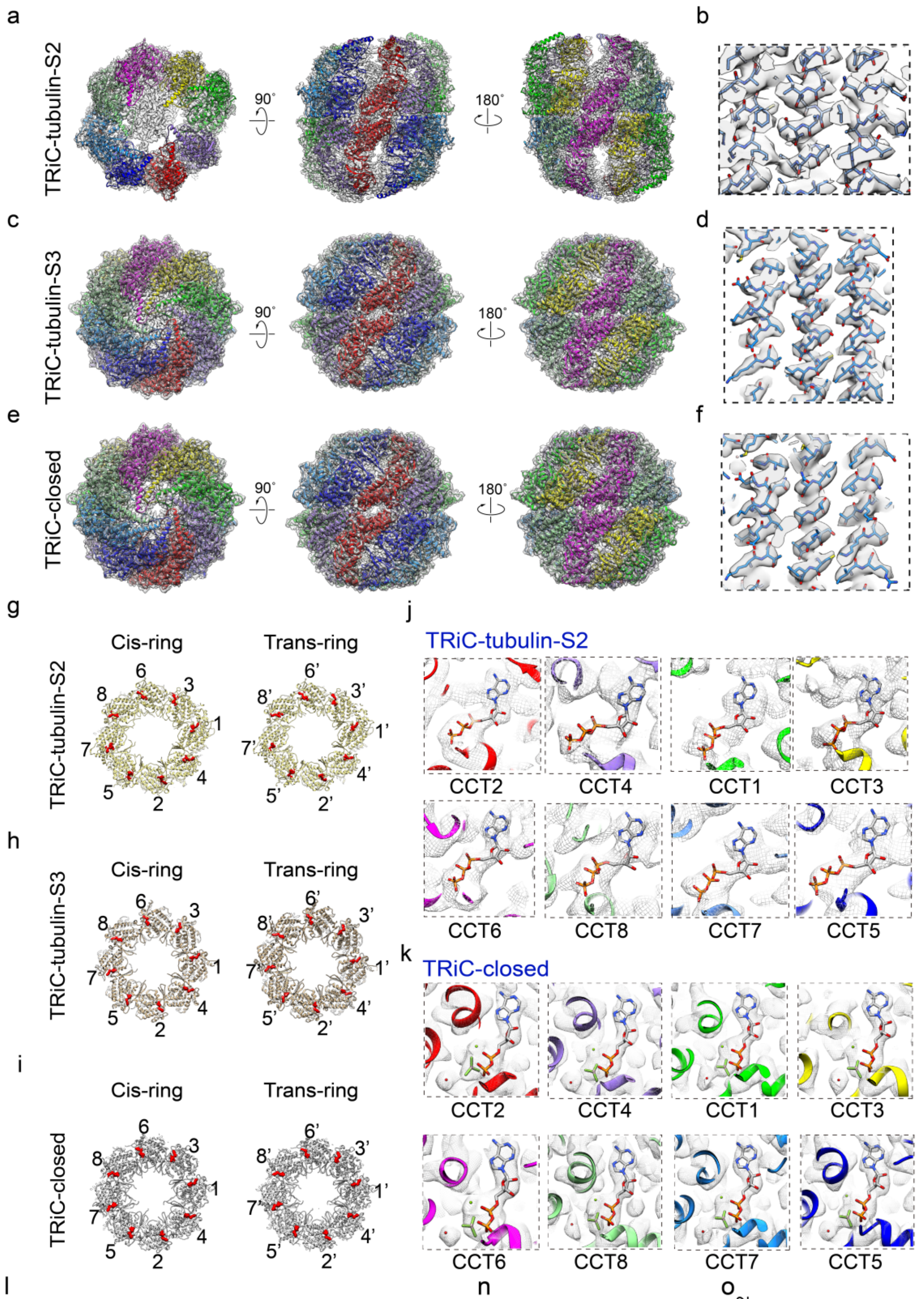

l

Tubulin-CCT	2	4	1	3	6	8	7	5	8'
Interaction Interface (Å²)	184	281	678	1361	1100	842	198	137	67

m

Protrusion Loop	Loop^H10	Loop^H9	C terminus	Stem loop
CCT1/3/4/5/6/7/8	CCT1/3/6/8	CCT3/6/8	CCT1/2/3/5/6/7/8	CCT3/6/8

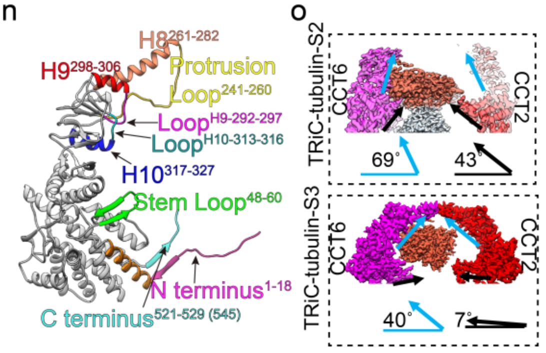

Supplementary Figure 7 | Atomic models of TRiC-tubulin-S2, -S3, TRiC-closed and nucleotide statuses in the S2 and TRiC-closed maps. **a,c,e** Model-map fitting of TRiC-tubulin-S2 (**a**), -S3 (**c**) and TRiC-closed (**e**), showing good matches between the models and maps. **b,d,f**, Representative high-resolution structural features of TRiC-tubulin-S2 (**b**), -S3 (**d**) and TRiC-closed (**f**). **g–i**, Nucleotide occupancy statuses of TRiC-tubulin-S2 (**g**), -S3 (**h**) and TRiC-closed (**i**). **j**, Magnified view of the TRiC-tubulin-S2 nucleotide pocket region, with every subunit appearing to include a bound nucleotide density matching the ATP model reasonably well, indicating that the TRiC-tubulin-S2 was in the ATP-bound state. **k**, Magnified view of the TRiC-closed nucleotide pocket region. Every subunit was found to be bound with ADP-AlFx (stick model), a magnesium ion (green ball), and a water molecule (red ball), suggesting that each of these subunits was in the ATP-hydrolysis transition state. **l**, Interaction areas between tubulin and TRiC subunits in TRiC-tubulin-S3 as calculated using PISA. **m**, Main structural elements of TRiC involved in the interaction with tubulin in TRiC-tubulin-S3. **n**, Key structural elements of TRiC involved in the interaction with tubulin, with the residue numbers of these elements labeled (CCT3 as a representative subunit). **o**, Depictions of a portion of the S2 and S3 maps (showing CCT6/2 subunits only), suggesting ATP-hydrolysis-induced downward rotations of TRiC A and E domains. In each panel, shown is the angle between the long axis of the A (in light blue) or E domain (in black) of CCT2 and the equator horizontal plane of the map.

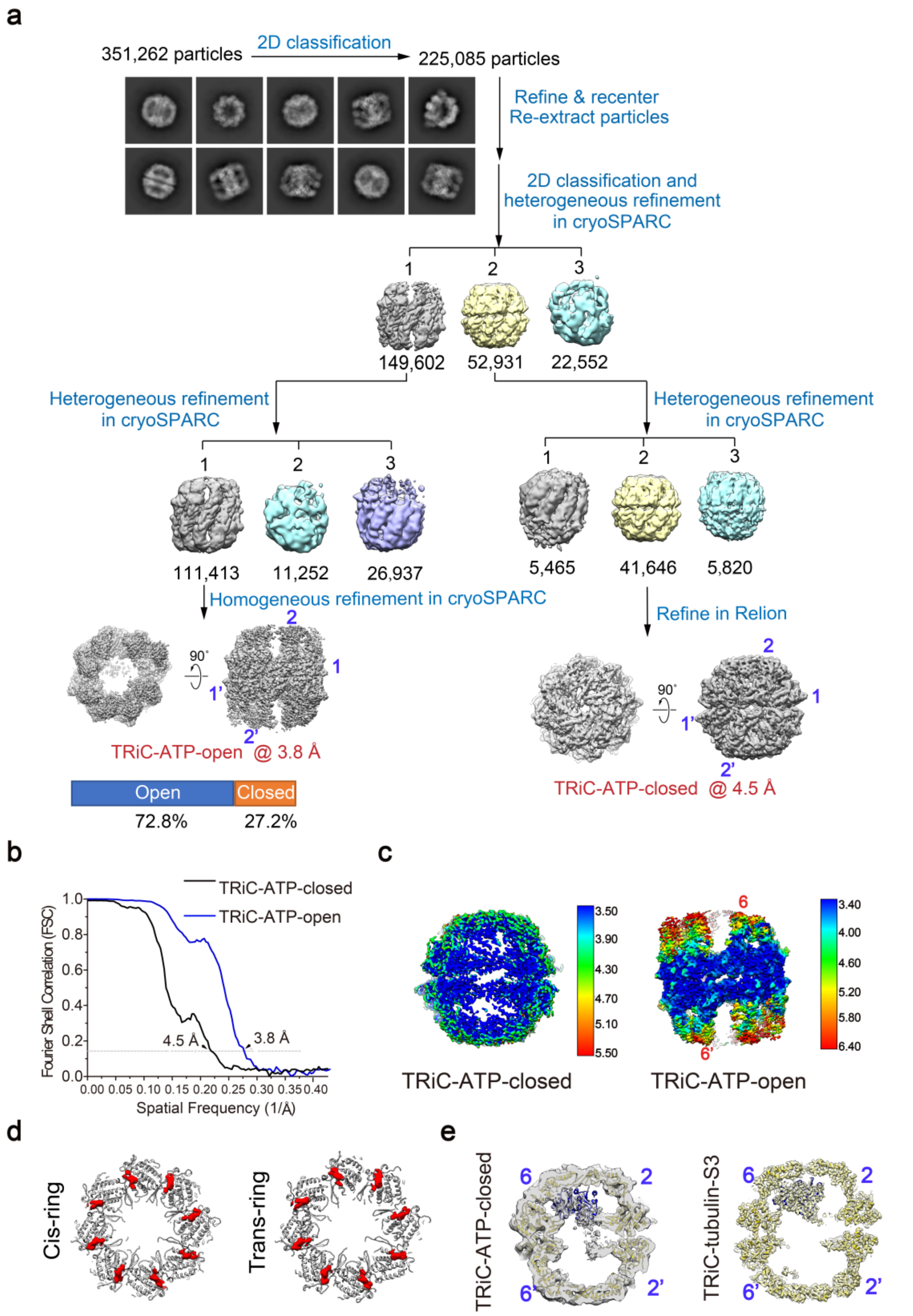

Supplementary Figure 8 | Cryo-EM analysis on TRiC in the presence of ATP. a, Work flow for the cryo-EM data processing of TRiC in the presence of ATP. Reference-free 2D class averages and population distributions of the TRiC-ATP-open and TRiC-ATP-closed were also presented. **b**,**c**, Resolution estimations according to the gold-

standard FSC criterion of 0.143 (b) and local resolution evaluations (c) for the TRiC-ATP-closed and TRiC-ATP-open maps. **d**, The nucleotide occupancy status of TRiC-ATP-closed, showing that all nucleotide pockets were occupied. **e**, Central slice views of the TRiC-ATP-closed (left, unsharpened) and TRiC-tubulin-S3 (right) maps with the fitted model of TRiC-tubulin-S3, indicating a substrate within one chamber of TRiC-ATP-closed, in a position and orientation similar to those of tubulin observed in the S3 map.

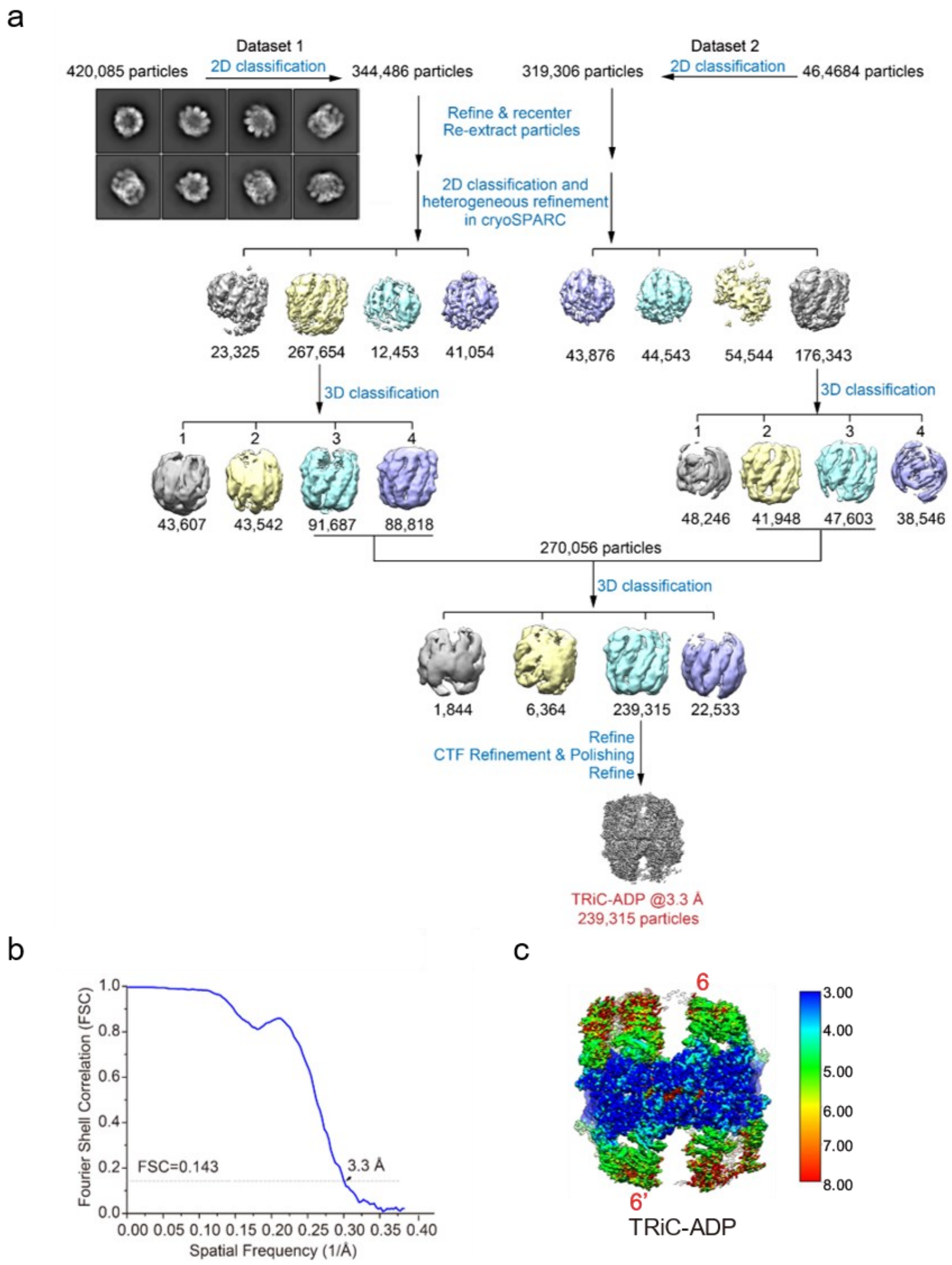

Supplementary Figure 9 | Cryo-EM analysis on the TRiC-ADP data. a, Work flow for the cryo-EM data processing of TRiC in the presence of ADP, with the reference-free 2D class average also shown. **b**, Estimation of the TRiC-ADP map resolution according to the gold-standard FSC criterion of 0.143. **c**, Local resolution evaluation for TRiC-ADP.

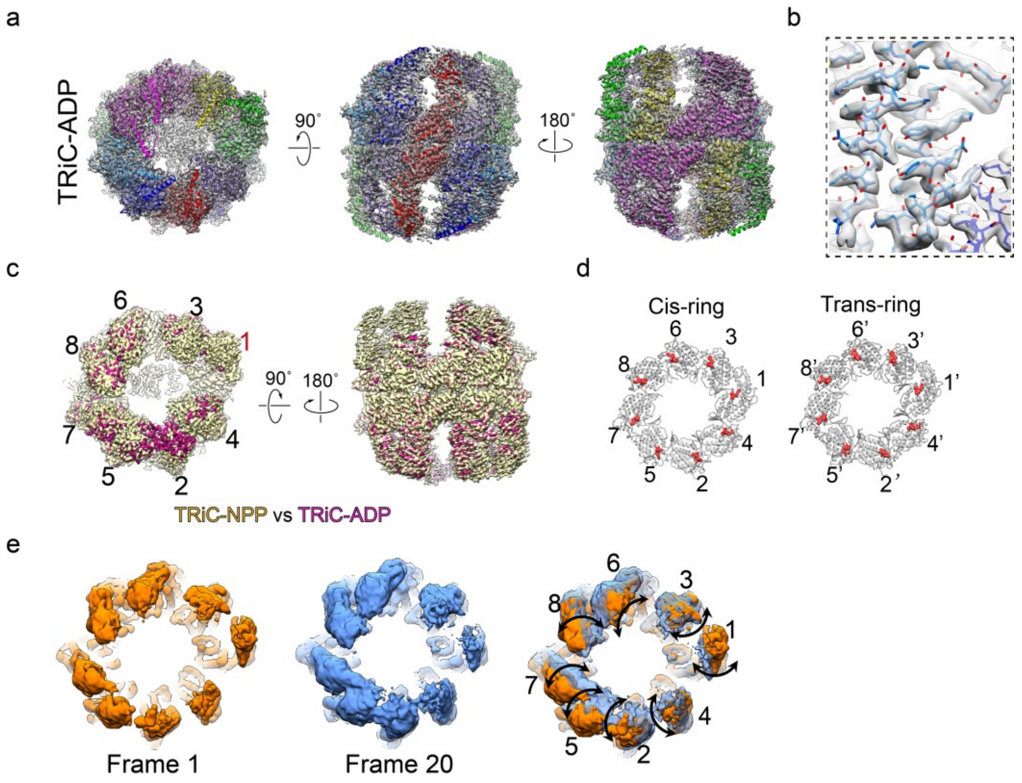

Supplementary Figure 10 | Atomic model and results of 3DVA of TRiC-ADP. a, Model and map fitting of TRiC-ADP, showing a good match between them. **b**, Representative high-resolution structural features of TRiC-ADP. **c**, Overlay of the TRiC-NPP (yellow) and TRiC-ADP (violet red) maps, indicating no obvious conformational differences between them. **d**, Structural depiction highlighting the determined nucleotide occupancy status of TRiC-ADP, showing that all the subunit nucleotide pockets were occupied. **e**, Results of a 3DVA of the TRiC-ADP dataset, suggesting that all of the subunits underwent outward/inward tilting motions. Frames 1 (orange) and 20 (cornflower blue), together showing the maximum extent of the conformational changes, are displayed.

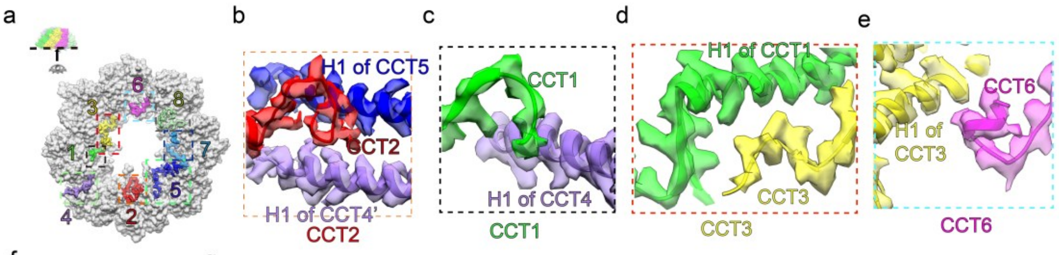

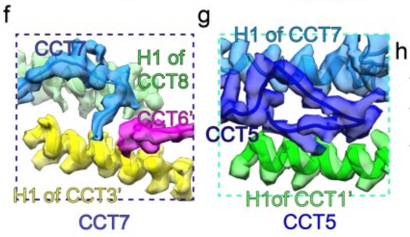

h

Subunit	CCT2	CCT4	CCT1	CCT3	CCT6	CCT8	CCT7	CCT5
A.A.	A7	K19	G3	H4	A2	H4	M1	T4

Supplementary Figure 11 | Involvement of the TRiC N-termini in the allosteric interaction network in TRiC-tubulin-S3. a, Locations of the resolved N-termini (in color) in the TRiC-tubulin-S3 structure. The visualization direction and visualized region are illustrated in the inset. **b–g**, Magnified views of the interaction between the N-terminus of CCT2 and helices H1 of CCT5 and CCT4' (**b**), between the N-terminus of CCT1 and H1 of CCT4 (**c**), between the N-terminus of CCT3 and H1 of CCT1 (**d**), between the CCT6 N-terminus (note the presence of a small 1-turn α-helix before its N-terminal β-sheet) and H1 of CCT3 (**e**), between the N-terminus of CCT7 and helices H1 of CCT8 and CCT3', as well as the N-terminus of CCT6' (**f**), and between the N-terminus of CCT5 and helices H1 of CCT7 and CCT1'(**g**). **h**, The most N-terminal amino acid residue visualized for each subunit of TRiC in the TRiC-tubulin-S3 structure.

Fig.1a, left, SDS-PAGE

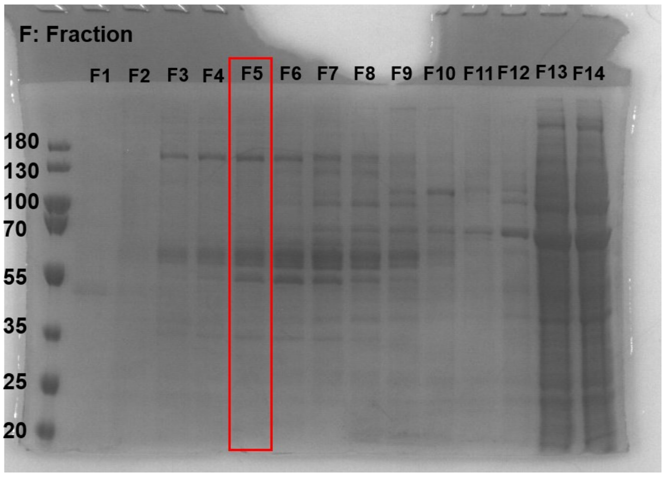

Fig. 1a, right, Anti-tubulin western blot

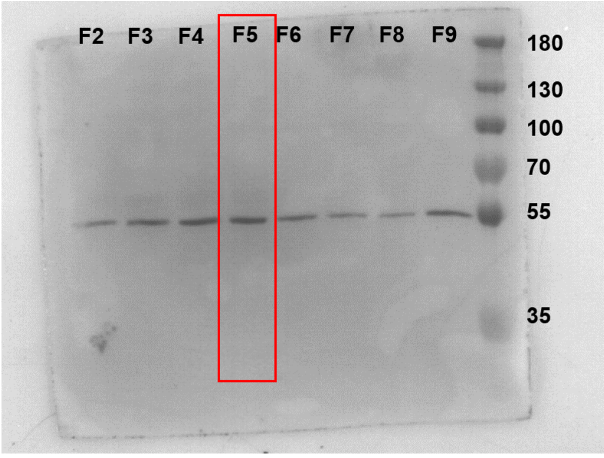

Fig. 1b, left, Native Page

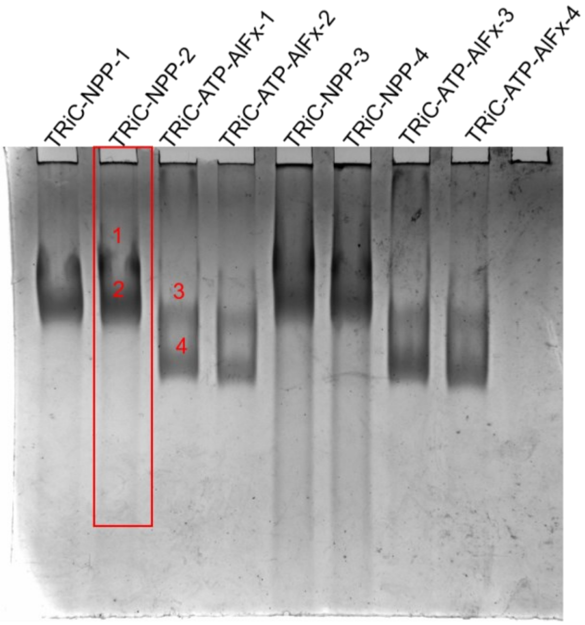

Fig.1b, upper right, SDS-PAGE, the number labeled here is consistent with the above panel

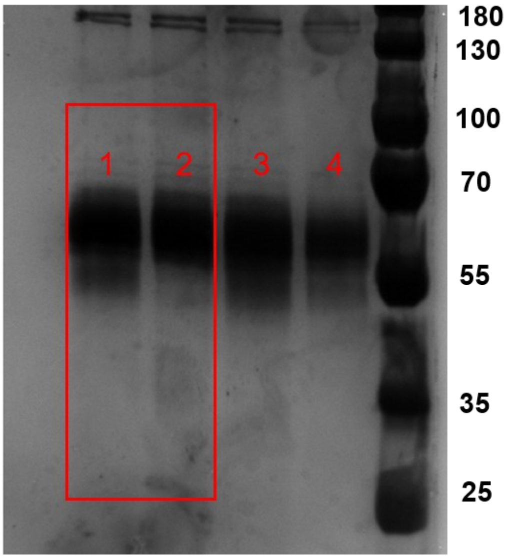

Fig.1b, lower right, western blot

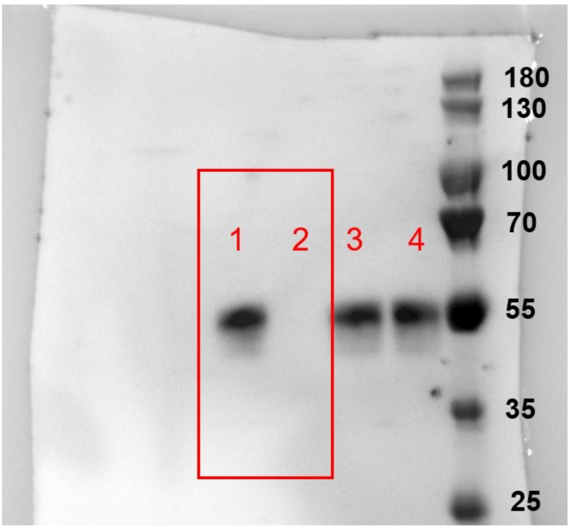

Supplementary Figure 12 | Uncropped images related to Fig. 1. The red rectangles represent the lanes shown in the figures.

Supplementary Table 1 | Results of mass spectroscopy (MS) analysis of endogenously purified TRiC with associated tubulin

	Protein	Coverage	PSMs	Peptides	Unique peptides
TRiC subunits	CCT1	79	116	39	37
	CCT2	78	199	36	36
	CCT3	73	113	41	41
	CCT4	72	125	33	32
	CCT5	68	139	41	39
	CCT6	64	124	35	30
	CCT7	80	194	39	32
	CCT8	75	121	40	40
TUBB	TUBB5	82	104	47	9
	TUBB4	38	31	16	3
	TUBB2	44	27	14	3
	TUBB6	24	12	7	2
	TUBA4	7	3	3	1
	TUBG1	24	7	6	6
	TUBD1	6	2	2	2

Supplementary Table 2 | Results of XL-MS analysis of TRiC with associated tubulin

Protein1 (site) –Protein2 (site) Inter-Protein	Peptides	Score	Spec count
CCT3 (21)-Tubulin (58)	KVQSGNINAAK(1)-ISVYYNEATGGKYVPR(12)	9.35E-09	3
CCT3 (21)-Tubulin (252)	KVQSGNINAAK(1)-KLAVNMVPFPR(1)	7.00E-10	4
CCT4 (21)-Tubulin (58)	GKGAYQDR(2)-ISVYYNEATGGKYVPR(12)	1.42E-22	3
CCT6 (10)-Tubulin (252)	TLNPKAEVAR(5)-KLAVNMVPFPR(1)	8.44E-07	4
CCT8 (16)-Tubulin (252)	APGFAQMLKEGAK(9)-KLAVNMVPFPR(1)	5.15E-06	2

* We used E-value (1.00E-02) and spec count (larger than 1) as the threshold to remove low-confidence XL-MS data.

Supplementary Table 3 | Results of XL-MS analysis of TRiC with associated tubulin in the presence of ATP-AlFx

Protein1 (site) –Protein2 (site) Inter-Protein	Peptides	E-value	Spec count
CCT1 (532)-Tubulin (252)	IDDLIKLHPESK(6)-KLAVNMVPFPR(1)	3.12E-04	3
CCT2 (250)-Tubulin (252)	IKIFGSR(2)-KLAVNMVPFPR(1)	5.03E-06	3
CCT2 (522)-Tubulin (252)	VDNIIKAAPR(6)-KLAVNMVPFPR(1)	2.24E-14	9
CCT2 (248)-Tubulin (252)	ILIANTGMDTDKIK(12)-KLAVNMVPFPR(1)	8.77E-05	3
CCT3 (21)-Tubulin (58)	KVQSGNINAAK(1)-ISVYYNEATGGKYVPR(12)	6.03E-08	3
CCT3 (527)-Tubulin (252)	IDDIVSGHKK(9)-KLAVNMVPFPR(1)	1.20E-10	5
CCT3 (21)-Tubulin (252)	KVQSGNINAAK(1)-KLAVNMVPFPR(1)	1.61E-10	4
CCT3 (248)-Tubulin (252)	IVLLDSSLEYKK(11)-KLAVNMVPFPR(1)	5.19E-05	3
CCT4 (2)-Tubulin (252)	PENVAPR(1)-KLAVNMVPFPR(1)	9.06E-11	8
CCT5 (263)-Tubulin (252)	HKLDVTSVEDYK(2)-KLAVNMVPFPR(1)	1.12E-12	4
CCT5 (257)-Tubulin (252)	IAILTCPFEPPKPK(12)-KLAVNMVPFPR(1)	1.55E-05	3
CCT6 (10)-Tubulin (252)	TLNPKAEVAR(5)-KLAVNMVPFPR(1)	2.37E-20	4
CCT7 (55)-Tubulin (252)	GKATISNDGATILK(2)-KLAVNMVPFPR(1)	1.01E-21	4
CCT8 (296)-Tubulin (252)	AIADTGANVVVTGGKVADMALHYANK(15)-KLAVNMVPFPR(1)	2.39E-10	8
CCT8 (225)-Tubulin (58)	KETEGDVTSVK(1)-ISVYYNEATGGKYVPR(12)	4.44E-08	3
CCT8 (225)-Tubulin (252)	KETEGDVTSVK(1)-KLAVNMVPFPR(1)	1.36E-08	2
CCT8 (318)-Tubulin (252)	LNSKWDLR(4)-KLAVNMVPFPR(1)	5.09E-13	5

* We used E-value (1.00E-02) and spec count (larger than 1) as the threshold to remove low-confidence XL-MS data.

Supplementary Table 4 | Interactions between tubulin and CCT3/6/8/1 subunits in the TRiC-tubulin-S3 structure detected using PISA

	TRiC subunit		Tubulin		Interaction	Distance (Å)
	Residue	Atom	Residue	Atom		
CCT3	GLU 89	[OE1]	LYS 392	[NZ]	Salt bridge/ H bond	3.82
	GLU 69	[OE2]	HIS 396	[NE2]	Salt bridge	3.32
	ARG 313	[NE]	GLU 157	[OE2]	Salt bridge	3.72
	ARG 200	[NH1]	ASP 404	[OD2]	Salt bridge/ H bond	3.65
	ARG 316	[NH1]	GLU 407	[OE2]	Salt bridge/ H bond	3.71
	LYS 317	[NZ]	GLU 410	[OE1]	Salt bridge/ H bond	2.52
	LYS 317	[NZ]	GLU 410	[OE2]	Salt bridge	3.87
	LYS 196	[NZ]	GLU 405	[OE2]	Salt bridge	3.90
	GLU 69	[OE1]	TRP 397	[NE1]	H bond	3.74
	GLU 69	[OE2]	TRP 397	[NE1]	H bond	3.64
	ARG 316	[NH1]	TYR 106	[OH]	H bond	2.48
	ARG 85	[NH1]	ARG 391	[O]	H bond	2.83
	ARG 68	[NH2]	LYS 392	[O]	H bond	3.74
	LYS 203	[NZ]	GLY 400	[O]	H bond	3.30
	ARG 200	[NH1]	MET 403	[O]	H bond	3.33
	ARG 200	[NH2]	MET 403	[O]	H bond	3.49
	ASN 321	[ND2]	MET 406	[SD]	H bond	3.26
	THR 318	[OG1]	GLU 407	[OE2]	H bond	3.03
CCT6	ASP 213	[OD1]	ARG 121	[NH1]	Salt bridge/ H bond	3.22
	ASP 213	[OD1]	ARG 121	[NH2]	Salt bridge	3.41
	ASP 213	[OD2]	LYS 122	[NZ]	Salt bridge/ H bond	2.45
	ARG 319	[NH1]	ASP 114	[OD2]	Salt bridge	3.49
	ARG 319	[NH2]	ASP 114	[OD2]	Salt bridge/ H bond	2.73
	ARG 314	[NH2]	ASP 128	[OD1]	Salt bridge/ H bond	3.17
	ARG 318	[NH1]	GLU 157	[OE1]	Salt bridge/ H bond	3.27
	GLU 357	[OE1]	THR 55	[OG1]	H bond	2.84
	ARG 315	[O]	ARG 121	[NH1]	H bond	3.28
	ASP 213	[OD2]	ARG 121	[NH2]	H bond	2.44
	TYR 239	[O]	ARG 162	[NH1]	H bond	3.48
	ALA 51	[N]	ASP 74	[OD2]	H bond	3.58
	ARG 314	[NH2]	GLU 125	[O]	H bond	2.48
	ARG 314	[NH1]	ASP 128	[O]	H bond	3.89
	ARG 217	[NE]	GLU 125	[O]	H bond	3.04
	GLY 215	[N]	GLU 125	[OE2]	H bond	3.79
	GLU 238	[OE1]	LEU 130	[N]	H bond	3.75
	GLN 294	[NE2]	GLU 158	[OE1]	H bond	2.69
CCT8	GLU 252	[OE1]	ARG 2	[NE]	Salt bridge/ H bond	3.27
	GLU 252	[OE1]	ARG 2	[NH2]	Salt bridge/ H bond	3.26
	ARG 314	[NE]	ASP 41	[OD2]	Salt bridge	3.69
	ARG 314	[NH1]	ASP 41	[OD1]	Salt bridge	3.51
	LYS 318	[NZ]	ASP 128	[OD2]	Salt bridge/ H bond	3.58
	GLU 252	[O]	ARG 2	[NH2]	H bond	2.98
	LYS 225	[NZ]	TYR 36	[OH]	H bond	3.67
	ARG 314	[NH1]	ASP 41	[OD2]	H bond	2.97
	ASN 316	[ND2]	ASP 41	[OD1]	H bond	2.68
	SER 317	[OG]	GLU 53	[OE2]	H bond	2.34
	LYS 318	[N]	GLU 53	[OE1]	H bond	3.28
	TRP 319	[NE1]	GLU 53	[O]	H bond	2.66
CCT1	ARG 309	[NH2]	GLU 410	[OE1]	Salt bridge/ H bond	3.66
	ASP 358	[O]	LYS 392	[NZ]	H bond	2.94
	GLN 242	[NE2]	ASP 417	[OD1]	H bond	3.81
	GLN 242	[OE1]	GLN 424	[NE2]	H bond	3.55
	ARG 310	[NH2]	ASN 416	[O]	H bond	3.31
	ARG 310	[NH2]	SER 420	[OG]	H bond	2.74

Supplementary Video Legends

Supplementary Video 1 | 3D variability analysis (3DVA) of the TRiC-NPP cryo-EM data. The 3DVA results suggested the A/I domains of the CCT1/4/2/5/7 subunits to be overall relatively dynamic, with those of CCT7/5/1 displaying even larger movements.

Supplementary Video 2 | 3D variability analysis (3DVA) of the TRiC-ADP cryo-EM data. The 3DVA results suggested all the TRiC subunits in TRiC-ADP to be very dynamic, including the usually relatively stable CCT6, with all subunits displaying an outward/inward tilting motion.
