# Supplementary material for: Pathway and mechanism of tubulin folding mediated by TRiC/CCT along its ATPase cycle revealed using cryo-EM
Source: Commun Biol. 2023 May 16;6:531. doi: 10.1038/s42003-023-04915-x (PMC10188570; doi:10.1038/s42003-023-04915-x)
Supplement: Supplementary file 3 — Description of Additional Supplementary Files [file 42003_2023_4915_MOESM3_ESM.pdf]

Description of Additional Supplementary Files

File name: Supplementary Video 1

Description: 3D variability analysis (3DVA) of the TRiC-NPP cryoEM data. The 3DVA results suggested the A/I domains of the CCT1/4/2/5/7 subunits to be overall relatively dynamic, with those of CCT7/5/1 displaying even larger movements.

File name: Supplementary Video 2

Description: 3D variability analysis (3DVA) of the TRiC-ADP cryoEM data. The 3DVA results suggested all the TRiC subunits in TRiC-ADP to be very dynamic, including the usually relatively stable CCT6, with all subunits displaying an outward/inward tilting motion.
